# Simultaneous spatiotemporal transcriptomics and microscopy of *Bacillus subtilis* swarm development reveal cooperation across generations

**DOI:** 10.1038/s41564-023-01518-4

**Published:** 2023-11-16

**Authors:** Hannah Jeckel, Kazuki Nosho, Konstantin Neuhaus, Alasdair D. Hastewell, Dominic J. Skinner, Dibya Saha, Niklas Netter, Nicole Paczia, Jörn Dunkel, Knut Drescher

**Affiliations:** 1 Biozentrum, University of Basel, Basel, Switzerland; 2 Department of Physics, Philipps-Universität Marburg, Marburg, Germany; 3 Department of Mathematics, Massachusetts Institute of Technology, Cambridge, MA USA; 4 NSF-Simons Center for Quantitative Biology, Northwestern University, Evanston, IL USA; 5 Max Planck Institute for Terrestrial Microbiology, Marburg, Germany

**Keywords:** Microbial communities, Data processing, Biofilms, Image processing, Cellular motility

## Abstract

Development of microbial communities is a complex multiscale phenomenon with wide-ranging biomedical and ecological implications. How biological and physical processes determine emergent spatial structures in microbial communities remains poorly understood due to a lack of simultaneous measurements of gene expression and cellular behaviour in space and time. Here we combined live-cell microscopy with a robotic arm for spatiotemporal sampling, which enabled us to simultaneously acquire phenotypic imaging data and spatiotemporal transcriptomes during *Bacillus subtilis* swarm development. Quantitative characterization of the spatiotemporal gene expression patterns revealed correlations with cellular and collective properties, and phenotypic subpopulations. By integrating these data with spatiotemporal metabolome measurements, we discovered a spatiotemporal cross-feeding mechanism fuelling swarm development: during their migration, earlier generations deposit metabolites which are consumed by later generations that swarm across the same location. These results highlight the importance of spatiotemporal effects during the emergence of phenotypic subpopulations and their interactions in bacterial communities.

## Abstract (web summary)

Spatiotemporal transcriptomes during multicellular development of *Bacillus subtilis* swarms reveal supra-generational cooperation.

## Main

Dynamically evolving microbial communities with spatial structure are ubiquitous in nature, from the intestinal microbiota in humans to soil-based biofilms and bacterial swarms expanding across moist surfaces^1–5^. Spatiotemporal patterns in microbial communities can emerge for the arrangement of genotypes, for phenotypic subpopulations of the same genotype and for emergent community properties, such as resource gradients, biophysical properties and stress tolerance^6–10^. Pattern formation in microbial communities and other multicellular systems is a complex multiscale process influenced by cellular growth, division, differentiation, motility as well as many types of chemical and physical cell–cell interactions. All these factors can change in space and time due to the varying resource availabilities within developing communities^11,12^. Even for the simplest microbial communities, such as single-species bacterial swarms, the number and spatiotemporal variability of the parameters influencing community development lead to a degree of complexity that makes it difficult to disentangle which intracellular processes and cellular interactions determine the emergent spatial structure of the community.

To understand how spatial structure arises during bacterial community development, multiscale spatiotemporal measurements of intracellular states, cellular phenotypes and multicellular structures are required. While advances in fluorescence microscopy and increasingly accurate image analysis tools have made it possible to simultaneously track single cells and the overall community structure in space and time^13–16^, methodologies for spatiotemporal measurements of intracellular states, such as transcriptomes, proteomes or metabolomes, are only beginning to emerge. Recently, fluorescence in situ hybridization techniques with sequential rounds of labelling and imaging have enabled ~100 simultaneous transcript levels to be detected in fixed bacterial communities^17^, and spatial mass spectrometry and Raman spectroscopy have enabled metabolite measurements in communities at single-cell resolution^18–21^. However, spatiotemporal omics methods for live measurements on developing communities are still lacking. Furthermore, suitable data analysis concepts are required for integrating different types of spatiotemporal data to connect the gene expression level to the cellular and multicellular phenotypes.

Using *Bacillus subtilis* swarm development as a model system for the emergence of spatial structure in multicellular communities, we developed an experimental platform for the measurement of spatiotemporal transcriptomes from live communities with high genome coverage, and the simultaneous acquisition of microscopy-based measurements of cellular phenotypes, multicellular phenotypes and the whole swarm development. By integrating these different levels of biological and biophysical information, we systematically uncovered spatiotemporally varying processes and properties of the swarm, and we identified different metabolic subpopulations within the swarm. Spatiotemporal measurements of metabolites that are secreted and consumed by the different subpopulations led us to discover spatiotemporal cross-feeding interactions within the swarm.

## Results

### Simultaneous live-cell imaging and robotic sampling during swarm development

Swarm development was monitored after inoculating *B. subtilis* NCIB 3610 cells onto a soft agar LB plate, which was incubated at 30 °C in a humidified chamber on a microscope. After an initial lag phase, during which the cells differentiated into the characteristic hyperflagellated swarmer cell type^22,23^, the swarm expanded from a diameter of 1 mm to 60 mm within ~6 h, as shown in Supplementary Movie 1. During swarm expansion, the swarm displayed circular symmetry at the macroscopic level (Supplementary Movie 1). At the microscopic level, spatially separated subpopulations with different cell shapes and motility behaviour emerge over time, eventually resulting in a three-dimensional (3D) biofilm in the swarm centre and a region of highly motile, collectively moving bacteria in the cell monolayer at the swarm front^23–27^.

**Fig. 1:**
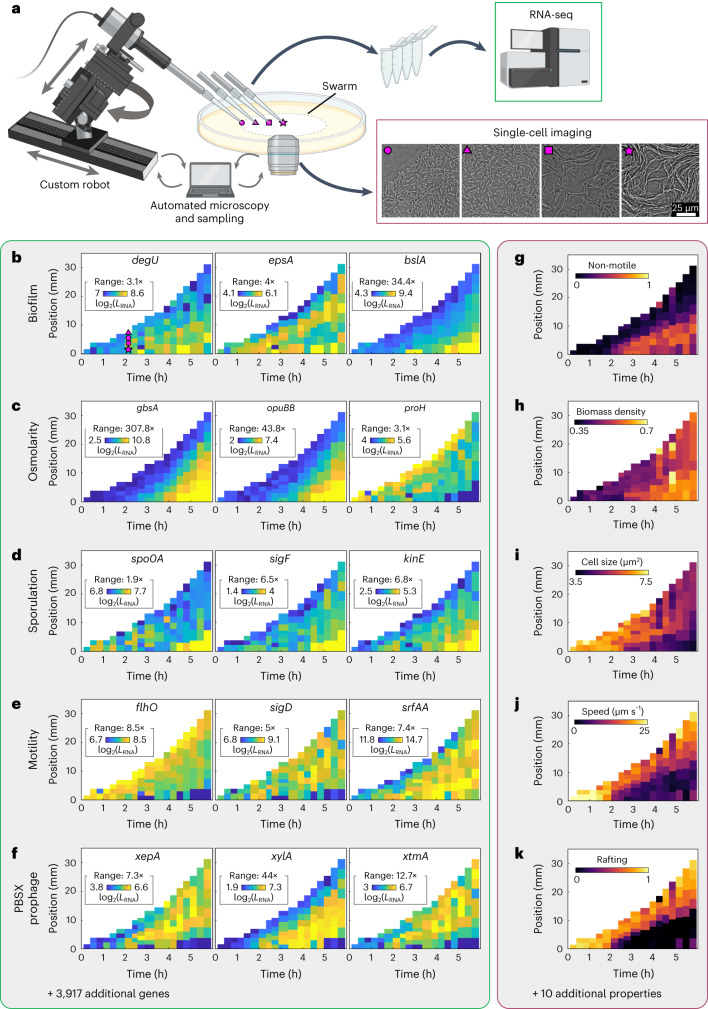
Spatiotemporal transcriptome measurements and microscopy-based phenotyping during *Bacillus subtilis* swarm development. **a**, A custom-built sampling robot is connected to an adaptive microscope, which enabled the synchronization of image acquisition and sampling of bacterial cells for transcriptome analysis. The robot collected cells from defined spatial and temporal locations within the developing swarm using custom-developed sampling tips. For each spatiotemporal sampling location, a brightfield microscopy video was acquired, depicting the behaviour of cells before their removal from the surface. Created using BioRender.com. **b**, Spatiotemporal transcriptome results are summarized in kymograph heat maps, with each coloured tile corresponding to one sample; the colour of each tile in the heat map indicates the expression level of a particular gene, *L*_RNA_. The ‘range’ value corresponds to the dynamic range of gene expression, defined as the ratio between the highest and lowest colour bar value, which are the 5th and 95th percentiles of the gene expression values, taking all three replicates into account (additional replicates shown in Supplementary Figs. 3–7). Three spatiotemporal heat maps depicting the expression pattern of genes related to biofilm development are shown: *degU*, *bslA*, *epsA*. **c**, Genes associated with osmoprotection: *gbsA*, *opuBB*, *proH*. **d**, Sporulation-associated genes: *spo0A*, *sigF*, *kinE*. **e**, Spatiotemporal gene expression heat maps for motility (*flhO*, *sigD*) and surfactin production (*srfAA*). **f**, PBSX prophage genes: *xepA*, *xylA*, *xtmA*. Thousands of additional spatiotemporal gene expression heat maps are available in our online dataset explorer. Replicates of genes displayed in **b**–**f** and other relevant genes are shown in Supplementary Figs. 3–21. **g**–**k**, Spatiotemporal phenotype heat maps, analogous to the gene expression heat maps in **b**–**f**. **g**, Ratio of non-motile cells to all detected cells in the field of view. **h**, Local biomass density, the mean fraction of area covered by cells in a circle around each cell´s centroid, is highest for late timepoints in the centre of the swarm. **i**, Average size of a *B. subtilis* cell. **j**, Average bacterial cell speed in space and time during swarm development. **k**, Rafting cells can be observed predominantly close to the swarm front. Similar spatiotemporal phenotype maps are available for 10 additional properties listed in Supplementary Table 2.

**Extended Data Fig. 1:**
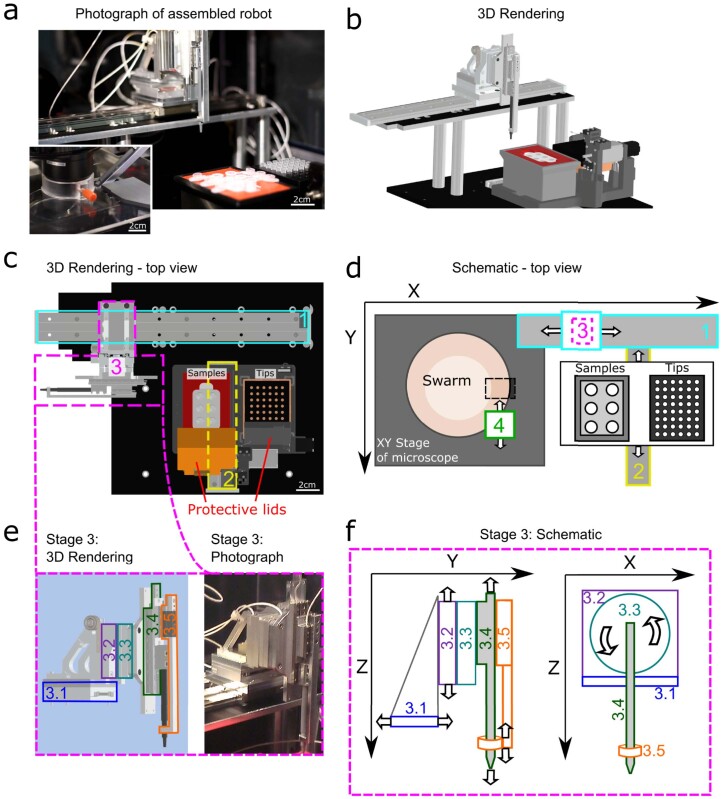
Overview of the robotic setup that was constructed to automatically extract cells with high spatiotemporal accuracy from the swarm. **(a)**, Photograph of the assembled robot, positioned on the right-hand side of the microscope objective. The inset photograph shows the robotic arm and sampling tip directly above the objective while it picks up cells from the swarm inside the Petri dish. The long horizontal stage in the central area of the main photograph (stage #1 in panel **d**) moves the robot back and forth between the sampling tips (bottom right), the petri dish containing the swarm placed on the microscope objective (left, outside of the image field of view, but shown in the inset photograph) and back to the sample ejection area (Eppendorf tubes within a cooled box in the central bottom part of the image, see also panel **d** for a schematic overview of the whole setup). Protective lids, which are placed on the tip box and sample ejection box were removed for the photograph to better show all components. The inset photograph also shows a transparent acrylic cover on top of the enclosure containing swarm to keep humidity high, with an opening for the sampling tip that is covered by a removable plastic plate (grey plate visible in the inset photograph), operated by a robotic stage (stage 4 in panel **d**). **(b)**, 3D rendering of the robot, using approximately the same viewing angle as in panel **a**, with the protective lids on the tip box and sample ejection box removed to better show the components. The 3D rendering files can be downloaded from the Zenodo repository (DOI: 10.5281/zenodo.8229225), in a format that can be viewed in the AutoDesk Inventor software; for convenience, the 3D rendering can be viewed online with full control of the viewing angle: https://gmail3493581.autodesk360.com/g/shares/SHd38bfQT1fb47330c99c60340a060ffa874. **(c)**, Top view of the 3D rendering, showing all components including protective lids. Robotic stages are labelled consistent with numeric labels in panel **d**. **(d)**, Schematic top view of the robotic setup, including the XY stage of the microscope. The robotic stages #1, #2, #4 refer to an individual stage, while #3 refers to a group of stages described further in panels **e** and **f**. **(e)**, Detailed 3D rendering view and photograph of the group of stages labelled #3 in panel **d**. This group of stages consists of several linear stages (stages 3.1, 3.2, 3.4, 3.5) and one rotational stage (stage 3.3). Attached to stage 3.4 is a tip holder (grey), which mimics the shape of the bottom part of a pipette, such that pipette tips can be picked up. Stage 3.5 mimics the ejection mechanism of a pipette, enabling automatic tip ejection. **(f)**, Schematic side views of the group of stages labelled #3 in panels **d** and **e**. How these stages are operated to perform sampling from the swarm is described in the *Methods* section.

**Extended Data Fig. 2:**
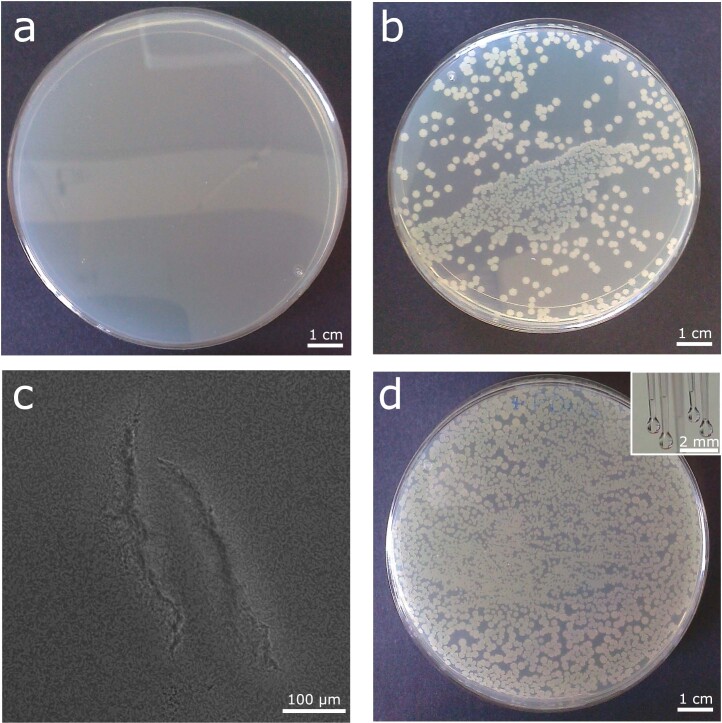
Sampling cells from the swarm using different methods. *B. subtilis* cells were collected from a swarm using a pipette tip and inoculated in 100 µL liquid LB medium prior to plating on fresh LB plates as seen in panels **a**, **b**, **d**. **(a)**, Cell extraction failed when using an unmodified commercial pipette tip to touch the surface of the swarm carefully without damaging the agar plate. **(b)**, When a sufficient number of cells was extracted by touching the swarm with an unmodified tip, the surface of the agar was scratched as can be seen in panel **(c)**. **(d)**, Using a modified tip, with a cured PDMS drop at the end, a large number of cells are acquired from the swarm, even when the surface is only carefully touched to not disrupt the agar surface. Inset: Tips are modified by dipping them into PDMS prior to incubation at 50 °C overnight in order to produce a small drop of cured PDMS.

**Extended Data Fig. 3:**
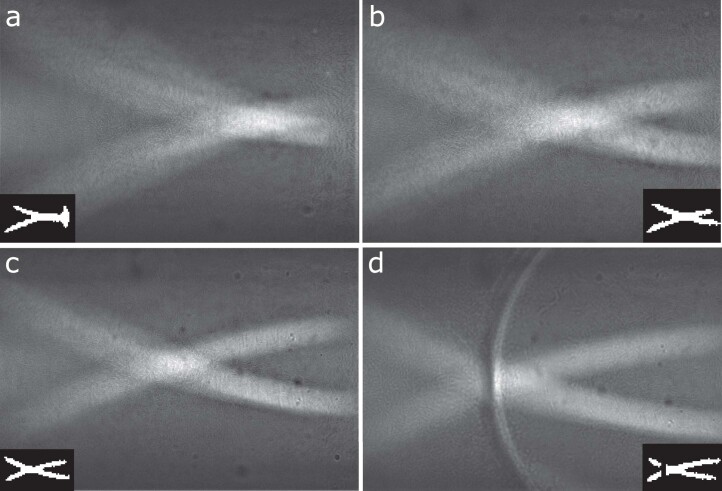
Guiding the tip of the robotic sampling arm by a distance-dependent light pattern and a feedback loop between image acquisition, live image analysis, and robotic arm control. To sample cells from the bacterial swarm, a sampling tip mounted on the robotic sampling arm is moved to the microscope as described in the Methods section and brought into position such that the tip is within the field of view of the microscope, approximately 1 mm above the swarming plate. At this point, a light pattern caused by the refraction of light through the PDMS drop at the end of the sampling tip can be observed through the camera. The shape of this pattern changes depending on the distance between the tip and the swarm surface and can therefore be used to guide the tip towards a careful pickup of cells. Images in panels a-d show different light patterns as viewed from the camera. Insets in each panel depict a binarization of the light patterns, obtained by thresholding, which was used for steering the robotic arm. The distances between the tip and agar surface are: **(a)**, 100 µm, **(b)**, 50 µm, **(c)**, 1 µm, **(d)**, 0 µm. The robotic arm is moved stepwise towards the agar surface, correcting the step size after each movement. Step sizes are chosen to be 50 µm, 30 µm, 20 µm, 10 µm, 2 µm or 1 µm, depending on the correlation of the current binarized image with two reference images representing the distances 100 µm and 1 µm. Step sizes are chosen several times smaller than the estimated distance to the surface to ensure that the tip will under no circumstances penetrate the agar further than 1 µm, which could lead to disruption of the agar surface and a disruption of the further swarm development. During the sampling process, the current image obtained from the microscope camera is displayed in a graphical user interface that allows manual intervention such as stopping the movement of the sampling tip as an additional layer of security. Upon making contact with the surface, the z-position of the point of contact is saved and used as a reference for future z-positioning of the sampling tip. The tip is then moved upwards and out of the humidity chamber before ejecting the sampling tip into an Eppendorf tube.

**Extended Data Fig. 4:**
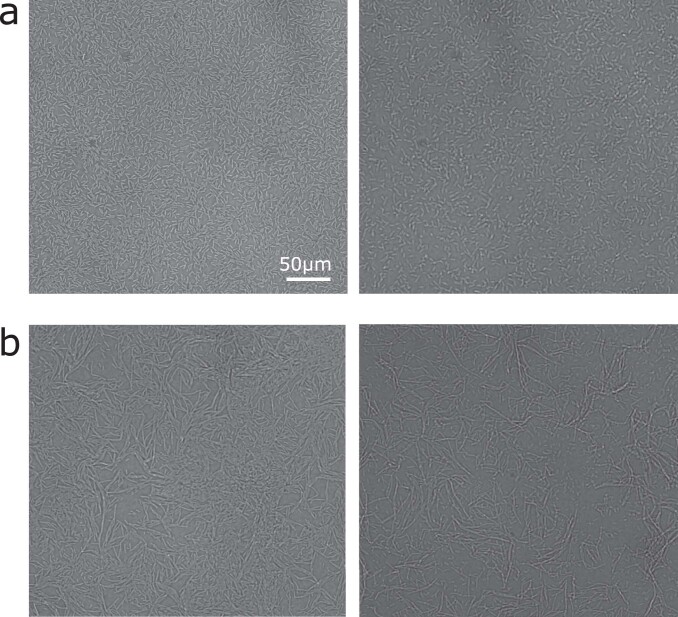
Microscopy images of bacterial swarm before and after sampling. To ensure that the sampling process was successfully completed, meaning that cells have been removed without damaging the agar, images are acquired before (left) and after (right) sampling at the same position. This data can also be used to estimate the number of bacterial cells which has been collected during sampling. **(a)** and **(b)** show two different example location of sampling.

**Extended Data Fig. 5:**
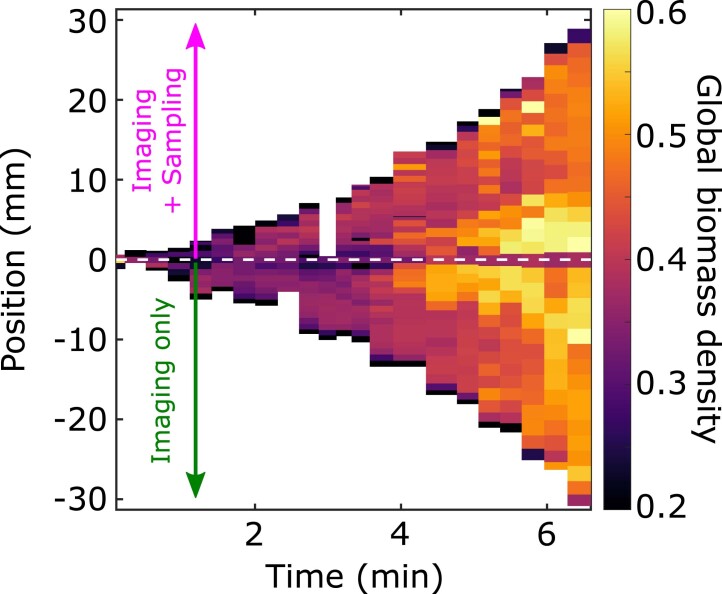
Spatiotemporal measurement of the “global biomass density” parameter during swarm development. Heatmap showing the spatiotemporal measurement results for the parameter “global biomass density” (see definition in Supplementary Table S2), based on microscopy and image analysis. The top half of the diagram represents the area of the swarm where both imaging and sampling of cells for transcriptome measurements took place, whereas the bottom half of the diagram represents the area where only imaging occurred. A comparison of these areas reveals that there is no significant difference in biomass density between the area from which cells are samples (top part) and the area where images are taken, but no sampling is performed (bottom part).

To simultaneously acquire spatiotemporal information of gene expression, cellular behaviour and multicellular dynamics during swarm expansion, we extended our previously developed adaptive microscope^23^ with a custom-built robotic sampling arm (Fig. 1a and Extended Data Fig. 1). The adaptive microscope enables us to acquire brightfield movies at single-cell resolution at a set of locations with varying radial position (*p*), at each stage of the swarm expansion. The software-controlled microscope adaptively expands the set of positions where movies are acquired depending on the swarm expansion, using a live feedback loop between image acquisition, image analysis and the motorized microscope. Immediately after a movie of the microscopic cell dynamics at a particular space–time location was acquired, the sampling tip of the robotic arm picked up 10^3^–10^5^ cells from the swarm at the same location, without disrupting the agar surface (see Supplementary Movie 2 and Extended Data Figs. 2–4). Cells collected on the tip were suspended directly in the lysis buffer and immediately frozen in liquid nitrogen until RNA isolation. This sample collection method enabled the isolation of intact RNA from a very low number of Gram-positive cells (~pg of mRNA), as the thawing process initiates cell lysis, which minimizes the incubation time for cell lysis and helps to preserve the RNA integrity. After quality controls and processing of isolated RNA to minimize potential sample-to-sample bias, we performed RNA-seq measurements that resulted in high genome coverage (3,932 distinct genes out of 4,342 possible genes in the genome and the plasmid pBS32 passed our detection limit (>10 reads in ${\ge}$2 samples)). Cells were only sampled from one half of the circularly symmetric swarm, while microscopy imaging was performed on both halves of the swarm. Microscopy observations showed that the reduction in cell density at the position from which cells were acquired recovered within ~15 min. Comparing the microscopy results of both halves of the swarm revealed that our sampling procedure did not alter the swarm development: both halves of the swarm expanded equally fast and the microscopic dynamics of positions imaged in both halves were indistinguishable (Extended Data Fig. 5 and Supplementary Figs. 1 and 2).

### Spatiotemporal phenotypic and transcriptomic characterization of swarm development

**Extended Data Fig. 6:**
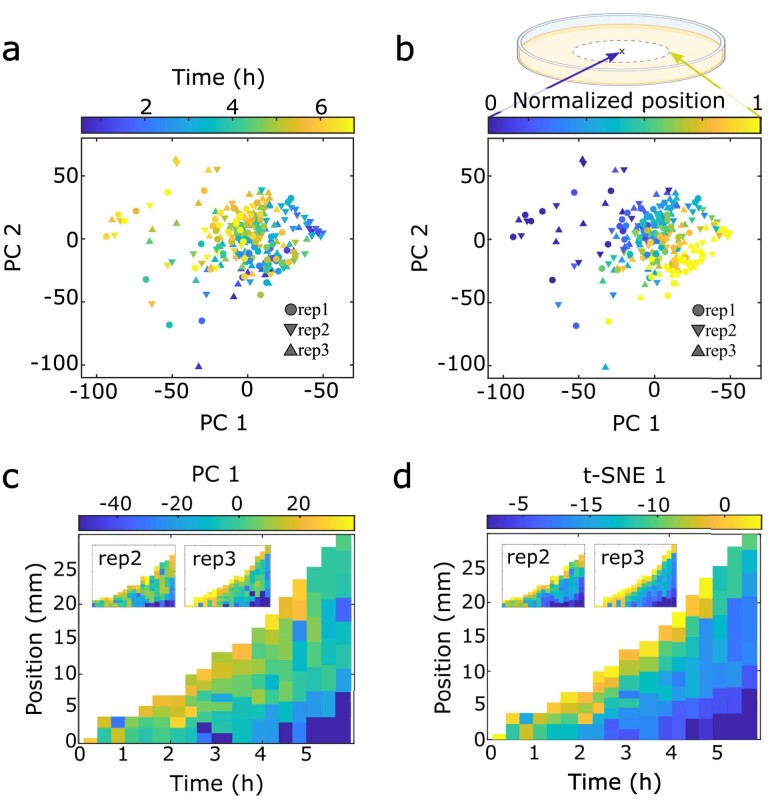
Low-dimensional representation of transcriptome data. **(a), (b)**, Applying principal component analysis to all transcriptomes of all biological replicates reveals that samples from different replicates (indicated by different symbols) are consistent with each other - each data point corresponds to one transcriptome. Furthermore, all samples and all replicates show the same trend when coloured by the sampling time **(a)** or normalized position **(b)**, which is defined by dividing the sample position by the current swarm radius. These observations show that the gene expression profiles are consistent across all replicates. Both the sampling time and normalized position correlate with principal component 1 (but with a stronger correlation for the normalized position). **(c)**, A spatiotemporal heatmap in which principal component 1 (PC 1, from panels a, b) is colour-coded shows that PC 1 gradually decreases from the swarm front towards the late swarm centre. **(d)**, A spatiotemporal heatmap in which t-SNE 1 is colour-coded shows that t-SNE 1 depends on space and time in the swarm, similar to PC 1, implying that both dimensionality reduction techniques identify consistent gene expression patterns. Taken together, the results presented in **(a)-(d)** show consistency across independent biological replicates and indicate that while the position of a sample has a strong influence on its gene expression profile, the sampling time also plays an important role and needs to be taken into account.

Using our adaptive microscope with the robotic sampling arm described above, we then obtained microscopic and macroscopic imaging data and simultaneously acquired cells for transcriptome measurements at ~100 defined spatial and temporal locations from a single developing swarm. We performed these measurements for *n* = 3 independent replicates of swarm development and found consistent results across the replicates. The spatiotemporal transcriptome and cellular dynamics data were visualized using heat maps, where each tile of a heat map corresponds to one spatiotemporal location for which a transcriptome (Fig. 1b–f) and microscopy video (Fig. 1g–k) were measured. Results from one biological replicate are shown in Fig. 1, and Supplementary Figs. 3–7 summarize corresponding measurements for the other biological replicates, as well as expression heat maps for genes from the same functional categories as those in Fig. 1. Spatiotemporal gene expression heat maps for genes related to additional biological processes that are relevant in biological communities are shown in Supplementary Figs. 8–21. A principal component analysis of the spatiotemporal transcriptome data revealed that the transcriptomes from different replicate experiments showed no systematic differences and that the transcriptome changes during swarm expansion were gradual without strongly separated clusters (Extended Data Fig. 6). The entire set of spatiotemporal heat maps for gene expression and microscopy-based measurements are available in a curated online data browser (https://drescherlab.org/data/swarm-transcriptome/) and we provide additional data formats as described in the data availability statement.

### Gene expression patterns change gradually in space and time and vary between different genes and processes

With our spatiotemporal transcriptome heat maps and the excellent bioinformatic tools that are available for *B. subtilis*^28,29^, we first inspected the expression of genes that are expected to play an important role in *B. subtilis* swarm development on the basis of previous studies^22,30–33^, such as genes important for biofilm formation (Fig. 1b), osmoregulation (Fig. 1c), sporulation (Fig. 1d) and motility (Fig. 1e). As expected, the occurrence of non-motile cells (Fig. 1g) closely correlates with the spatiotemporal expression pattern of the biofilm regulator *degU*. Furthermore, the expressions of the biofilm matrix genes *epsA* and *bslA* follow a hierarchy whereby the *eps* operon is expressed earlier than *bslA* during biofilm formation, which starts in the centre of the swarm already at the beginning of the swarm expansion phase and spreads outwards (Fig. 1b). We also observed strong spatiotemporal expression patterns for osmoregulatory genes: the heat maps for *gbsA* and *opuBB* indicate a highly increased production of the compatible solute glycine betaine in the late centre of the swarm (Fig. 1c), which coincides with the locations of high cell density (Fig. 1h). However, the spatiotemporal pattern of *proH* expression, which can be used as a reporter for osmotic stress^34^, implies that cells only experience weak osmotic stress in the late outer region of the swarm and not in the region where biofilms are formed (Fig. 1c). Sporulation genes are expressed primarily in the late centre of the swarm, which coincides with the region that displays biofilm formation (Fig. 1d), and small cell sizes (Fig. 1i). The spatiotemporal expression patterns of genes that are important for biofilm formation and sporulation are therefore roughly consistent with expectations.

Surprisingly, the spatiotemporal expression patterns of motility genes and surfactin production (Fig. 1e) only weakly correlate with the actual bacterial motility speed (Fig. 1j), indicating that additional factors beyond motility and surfactin gene expression are important for swarming. In previous work we showed that physical cell–cell interactions influence the microscopic behaviour of cells^23^, leading to raft formation in dense and motile regions, which enhances the individual cell speed. In contrast, subpopulations of non-motile elongated cells or cell chains producing matrix components^23,35^ are present in some regions of the swarm (Fig. 1g), which can act as obstacles that slow down motile cells. These mechanisms of increasing or decreasing cell speed are independent of motility gene expression and may therefore contribute to the qualitative difference between cell speed and motility gene expression patterns. Post-transcriptional regulation of flagella production and flagella activity^36^ can potentially also contribute to the observed qualitative difference between motility phenotype and gene expression. Temporally resolved measurements of transcriptomes during the lag phase (Supplementary Fig. 21), which precedes the swarm expansion phase shown in Fig. 1, revealed that for the surfactin synthesis operon, the largest regulation occurs during the lag phase. The lag phase transcriptomes also showed that the regulation of flagella assembly genes during swarm development mainly occurs in terms of an upregulation during the lag phase, followed by a spatiotemporally varying downregulation during the expansion phase (Supplementary Fig. 21).

Beyond the genes that are intuitively expected to be involved in swarm development (motility, biofilm, sporulation and osmoregulation genes, as described above), we noticed that many more genes gradually varied in space and time from the late centre to the front of the swarm, and we also observed that many genes did not display any spatiotemporal pattern. Furthermore, we noticed that several genes that were previously not implicated in swarm development, such as genes coding for the PBSX prophage, displayed a different spatiotemporal pattern (Fig. 1f): these genes were expressed at low levels in the late centre of the swarm and at low levels at the front of the swarm, but at high levels in the intermediate region. A similar spatiotemporal pattern, with high expression in the intermediate region and low expression in the centre and at the front, was also displayed by genes that are important for fatty acid synthesis (*fabD*, *fabHA, fabHB*, Supplementary Fig. 9) and surfactin production (*srfAA-AD*, Supplementary Fig. 10). Similarly, some phenotypic patterns, such as the fraction of cells in co-moving groups of cells (termed rafts, Fig. 1k), and the biomass density fluctuations (Supplementary Fig. 2) also displayed a distinct behaviour in the intermediate region between the swarm centre and the swarm front. The spatiotemporal gene expression and phenotypic patterns are therefore not always simple patterns that vary monotonically from the late centre of the swarm to the front of the swarm, and a more comprehensive analysis of the 3 ×3,932 gene expression heat maps and 3 ×15 phenotypic heat maps is required to systematically identify spatiotemporal patterns and pathways.

### Computational analysis identifies distinct spatiotemporal gene expression patterns

**Fig. 2:**
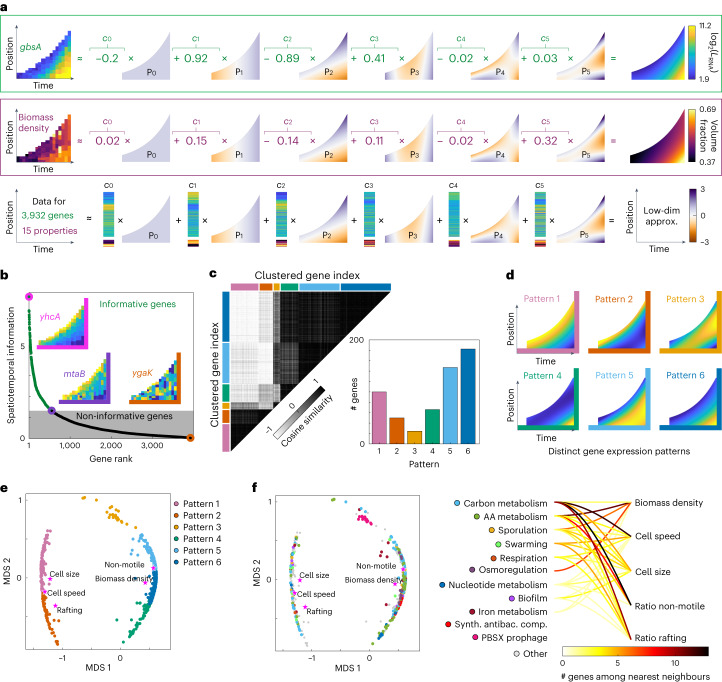
Identification of genes with spatiotemporal regulation and the different types of spatiotemporal expression pattern. **a**, The spatiotemporal expression heat map for each gene and each phenotypic property was approximated by a linear combination of six orthogonal basis functions *P*_*i*_ (*i* = 0,…,5) that are optimized for the spatiotemporal swarm domain. The coefficients ${c}_{i}$ of the basis functions were determined using all 3 biological replicates. **b**, A spatiotemporal information score was defined (Methods), which quantifies the level of spatiotemporal information observed for a given gene. By ranking genes according to this spatiotemporal information score and defining a cut-off at the weighted median of the spatiotemporal information (Methods), we identified all genes with spatiotemporal gene expression patterns. Insets show heat maps of gene expression with low, intermediate and high spatiotemporal information scores. **c**, For the 572 genes with spatiotemporal gene expression patterns identified in **b**, we used cosine similarity on the basis of the coefficients ${c}_{i}$ to identify clusters of highly correlated spatiotemporal patterns, revealing 6 major distinct spatiotemporal gene expression patterns (indicated by coloured bars on the right of the similarity matrix). **d**, To visualize these, the pattern corresponding to the mean of all coefficients ${c}_{i}$ within each pattern cluster is shown. **e**,**f**, Multidimensional scaling (MDS) was applied to genes and swarm properties on the basis of their cosine similarity shown in **c**. The expression pattern of each gene is represented as a point, with colour indicating their expression pattern category (**e**) or gene function (**f**). Gene functions are based on subtiWiki^28,29^. Gene function categories with fewer than 10 genes assigned to them are grouped into the category ‘other’. Five phenotypic properties of the swarm (see Fig. 1 for heat maps) are shown as stars, revealing that their location in the MDS space is in proximity to some gene patterns and functions. **f**, For each of these five phenotypic properties, the 50 nearest neighbour genes in the MDS space (corresponding to similar gene expression pattern) were identified and grouped into gene function categories. The number of genes in each gene function category for each phenotypic property is visualized in the connection plot (right).

To systematically characterize the different types of spatiotemporal patterns in gene expression and phenotypes during swarm development, we performed an unbiased analysis of the spatiotemporal data. For this, each spatiotemporal swarming dataset was represented by the coefficients ${c}_{i}$ of the orthogonal basis functions ${P}_{i}$ (Fig. 2a), which were tailored for the specific spatiotemporal swarming domain. Using the coefficients ${c}_{i}$ for each spatiotemporal dataset, we comprehensively compared how strongly each gene varies in space and time during swarming (Fig. 2b). For the 572 genes that displayed a high degree of spatiotemporal variation during swarming, we computed the similarity in their spatiotemporal expression patterns, which revealed 6 clusters of genes with different spatiotemporal expression patterns (Fig. 2c, inset shows the number of genes with a particular pattern). To illustrate the 6 different patterns, the pattern corresponding to the mean of all coefficients is shown in Fig. 2d. Interestingly, all 6 patterns vary in space and time, and not only in space or time.

### Spatiotemporal phenotype patterns correlate with metabolism gene expression patterns

To reveal connections between our measurements of gene expression and microscopy-based phenotypic properties, we performed a 2D embedding using multidimensional scaling for the spatiotemporally expressed genes and the phenotypic properties (Fig. 2e,f). This analysis shows that the biomass density and the abundance of non-motile cells in the swarm are closely related and are close to patterns 5 and 6, whereas motility-related phenotypic properties, such as the cell speed, the abundance of rafting cells and the cell size are close to patterns 1 and 2 (Fig. 2). By looking at the gene functions of the 50 closest genes to the phenotypic properties in Fig. 2f, we found that metabolic genes are most closely associated with the spatiotemporal dynamics of the phenotypic properties (Fig. 2f right). This led us to investigate the spatiotemporal changes in metabolism during swarm development, with the aim of understanding how and why metabolic changes occur in space and time, and how they influence swarm development.

### Different metabolic states emerge in different spatiotemporal regions of the swarm

**Fig. 3:**
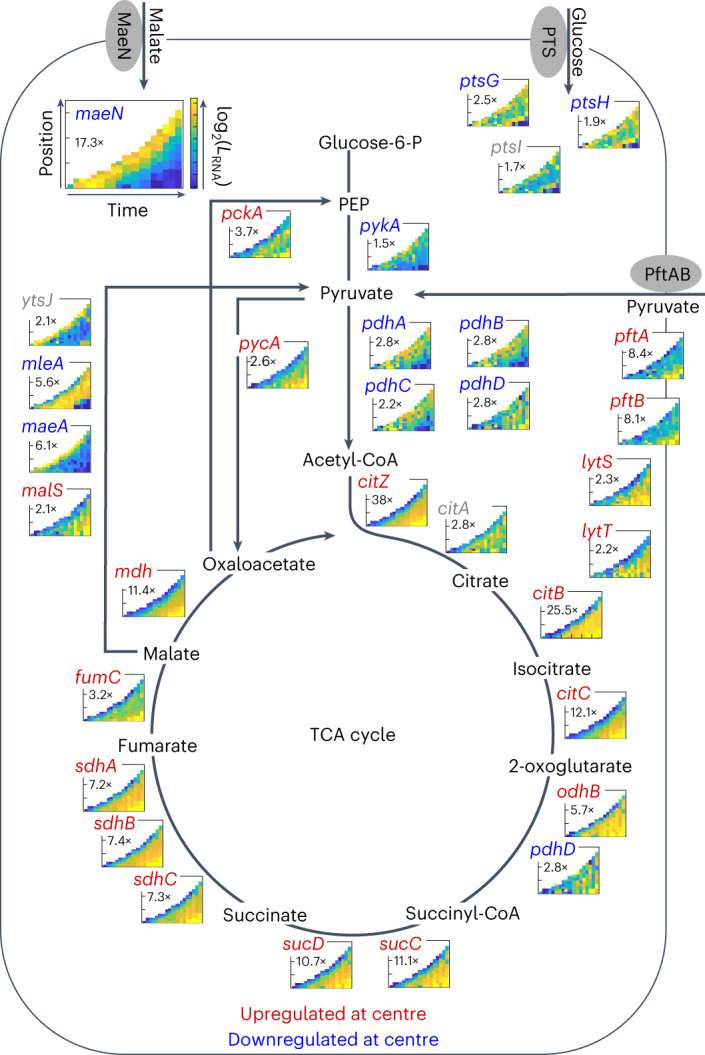
Spatiotemporal expression of core carbon metabolism genes during swarm development. Expression levels of genes are shown as space–time heat maps, analogous to the heat maps introduced in Fig. 1. The colour of the gene names indicates the regulatory pattern: red font indicates that the gene is upregulated in the late centre of the swarm, blue font indicates downregulation in the late swarm centre, and grey font indicates no clear expression pattern. The number below the gene name on each heat map indicates the dynamic range in gene expression between the maximum and minimum value in the heat map, analogous to the range values in Fig. 1. The genes are arranged according to a schematic representation of glycolysis and the TCA cycle, with an emphasis on malate processing. Genes coding for the main importer systems of malate (*maeN*) and glucose (PTS system) are shown at the top.

**Fig. 4:**
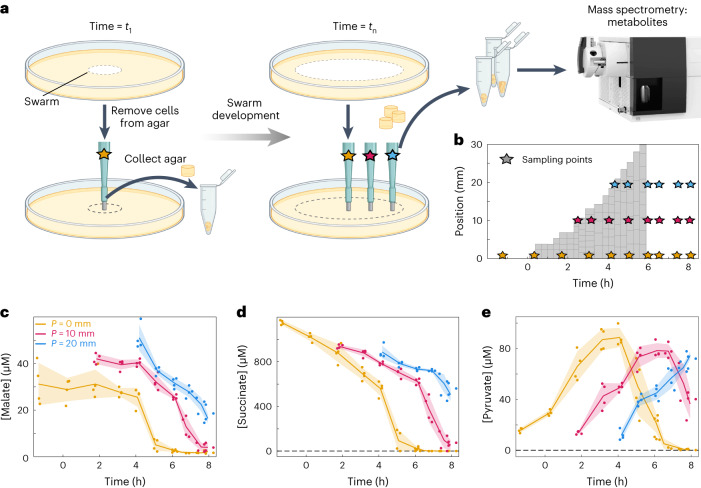
Extracellular metabolite measurements reveal spatiotemporal cross-feeding of pyruvate. **a**, To measure extracellular metabolites, cells were removed from the swarming plate before extracting a small piece of agar from up to 3 positions indicated by stars in different colours (orange position: *p* = 0 mm, pink position: *p* = 10 mm, blue position: *p* = 20 mm). Positions *p* were measured as radial distances from the point of inoculation. Similar samples were acquired for swarms that were grown for different times to measure extracellular metabolites at 3 spatial locations over time. Metabolites were extracted from the agar and measured using mass spectrometry. Created using BioRender.com. **b**, In comparison to the RNA-seq measurements (grey tiles), sampling points for extracellular metabolites (coloured stars) were sparser in space but were acquired for a longer period of time. **c**,**d**, Extracellular malate and succinate concentrations in the agar decreased to zero with increasing time, indicating that these compounds were consumed by the cells and eventually depleted. Colours indicate the 3 different sampling positions as described in **a**. **e**, The concentration of pyruvate increased over time before decreasing again, indicating that this metabolite was deposited by cells growing in malate- and succinate-rich medium, and later consumed by daughter cells as well as cells that have migrated to this position. Pyruvate is therefore cross-fed in space and time. In **c**–**e**, dots represent acquired samples, lines and shaded regions correspond to the mean ± s.d. calculated using *n* = 4 biologically independent samples in all cases except for the last timepoint, where *n* = 3 biologically independent samples were used.

As glucose and malate are the preferred carbon sources of *B. subtilis*^37^, we studied the spatiotemporal organization of glucose and malate utilization (Fig. 3). Glucose is only present at very low levels in LB^38^, and consequently we found that glucose uptake genes such as *ptsG* and *ptsH* have a weak spatiotemporal expression pattern and relatively low fold-change between minimum and maximum expression levels (Fig. 3 top right, additional replicates in Supplementary Fig. 11). In contrast, genes related to malate uptake and its utilization are strongly differentially regulated in space and time (Fig. 3 top left and Supplementary Fig. 12), which is consistent with our measurements of malate concentrations in LB, described further below and in Fig. 4. The malate transporter gene *maeN* is expressed 14-fold higher at the front compared with the swarm centre (Fig. 3 top left). Among genes coding malic enzymes that convert malate to pyruvate, *maeA*, *ytsJ* and *mleA* are highly expressed at the swarm front or in the intermediate region, although one malic enzyme-coding gene, *malS*, has an opposite pattern with a slightly higher expression at the late centre of the swarm (Fig. 3 left). Our transcriptome data indicate that at the swarm front, where cells experience a microenvironment with unconsumed LB medium, malate is taken up and processed into pyruvate, coupled with gluconeogenesis and glycolysis reactions to obtain energy via substrate-level phosphorylation despite the presence of oxygen (Supplementary Fig. 13). Previous measurements in liquid culture conditions with malate as the sole carbon source and in the presence of oxygen have shown that following malate uptake, excess pyruvate is secreted^37^.

In contrast to malate uptake, genes involved in the tricarboxylic acid (TCA) cycle mostly show a stronger expression at the late swarm centre (Fig. 3 bottom and Supplementary Fig. 14). For example, *citZ*, which is often the rate-limiting gene of the TCA cycle^39,40^, displays a 38-fold higher expression in the swarm centre compared with the swarm front (Fig. 3). These data indicate that cells at the swarm centre perform different metabolism than the cells at the swarm front: cells at the swarm centre produce energy mainly by running the TCA cycle and aerobic respiration (Supplementary Fig. 15). Interestingly, we noticed that the genes involved in pyruvate uptake, particularly the transporter *pftAB*^41,42^, and to a lower degree also the two-component system *lytST* are upregulated in the swarm centre (Fig. 3 right and Supplementary Fig. 16), similar to the TCA cycle genes. On the basis of this observation, and the speculation that pyruvate is probably secreted at the swarm front after malate uptake and utilization, we hypothesized that the following spatiotemporal metabolic interaction could take place within the developing swarm: cells at the swarm front rapidly consume malate and probably secrete pyruvate and other fermentation metabolites (Supplementary Fig. 17); these secreted metabolites would remain in the agar across which the swarm expands, and the next generation of cells that moves across the same location in the agar can potentially use these metabolites to fuel the TCA cycle.

### Spatiotemporal metabolome measurements reveal spatiotemporal cross-feeding

**Extended Data Fig. 7:**
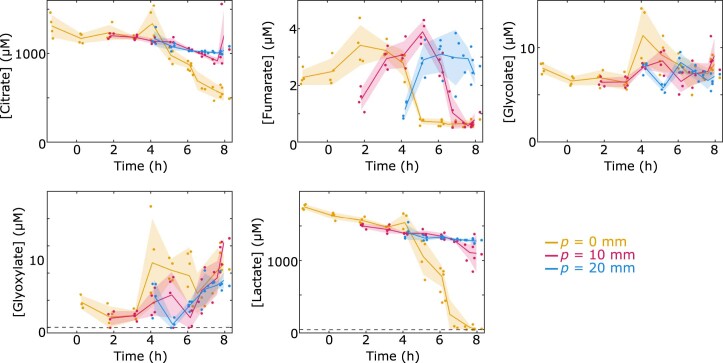
Measurements of organic acid concentrations in the agar underneath the swarm. Measurements for the compounds glycolate, glyoxylate, lactate, citrate and fumarate in the agar underneath the swarm, analogous to Fig. 4. Colours represent three different positions, analogous to Fig. 4. The measurement for each acquired sample is represented by a dot, lines correspond to the mean, and shaded regions indicate standard deviations. Means and standard deviations were calculated using n = 4 biologically independent samples in all cases except for the last timepoint, where n = 3 biologically independent samples were used.

To directly test this hypothesis, we performed additional experiments to measure the spatiotemporal concentration of extracellular metabolites in the agar beneath the developing swarm. For these experiments, swarms were grown for different durations (10 timepoints) before carefully removing all cells from the agar surface and extracting metabolites from a small amount of agar from up to 3 different spatial positions (Fig. 4a,b) for *n* = 4 independent replicate experiments. After a metabolite extraction process (see Methods), metabolites were measured with a targeted approach via mass spectrometry. The results for several metabolites are shown in Fig. 4c–e, and measurements for additional compounds are shown in Extended Data Fig. 7.

**Extended Data Fig. 8:**
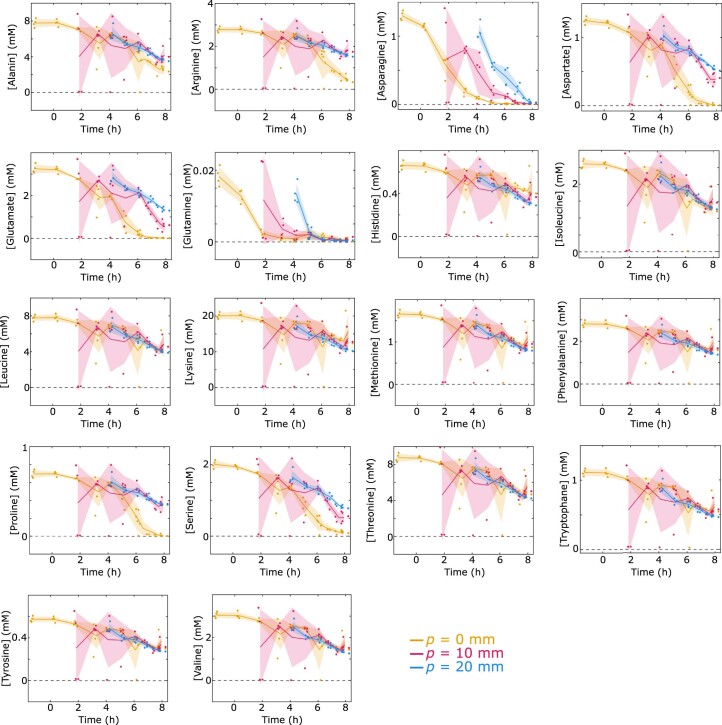
Measurements of amino acid concentrations in the agar underneath the swarm. The Concentration over time was measured for several amino acids. The three different colours correspond to three different positions in the swarm, analogous to Fig. 4. Glycine was measured, but could not be detected in any sample. The measurement for each acquired sample is represented by a dot, and lines correspond to the mean, and shaded regions indicate standard deviations. Means and standard deviations were calculated using n = 4 biologically independent samples in all cases except for the last timepoint, where n = 3 biologically independent samples were used.

The extracellular metabolite concentration time series at the three different spatial positions within the swarm revealed that some carbon sources are consumed until they are depleted, as the cells move across the spatial locations: initially the cells consume succinate (Fig. 4c), then malate (Fig. 4d) and several amino acids (see Extended Data Fig. 8 for amino acid measurements, and Supplementary Fig. 18 for amino acid uptake and synthesis gene expression). The extracellular pyruvate concentration, however, displays a different pattern. Initially, there is a very low concentration of pyruvate present in the agar before it is colonized. At very early times during swarm expansion, cells at the swarm front (which is very close to the swarm centre at these times) secrete pyruvate, which builds up at the swarm centre until it is consumed again at later times (Fig. 4e). The same pattern of pyruvate secretion followed by consumption then occurs with a time shift of ~2 h at the other spatial positions, which are separated from each other by 10 mm (Fig. 4e). The timepoint at which the concentration of pyruvate starts to decline coincides with the depletion of malate and succinate, indicating that pyruvate is only consumed once the other nutrients are becoming scarce. A similar pattern of metabolite secretion followed by consumption, with a 2-h time shift at different spatial locations, also occurs for fumarate (Extended Data Fig. 7). Diffusion of pyruvate or fumarate over the 10 mm distance between sampling positions can be estimated to take ~7 h (significantly longer than the observed 2-h time shift for the concentration profiles), and there is a pattern of diverging concentrations around *t* = 5 h, indicating that diffusion cannot be the dominant mechanism for determining the pyruvate and fumarate concentration dynamics. Instead, secretion and consumption of these metabolites are plausible explanations for causing the concentration dynamics.

Taken together, these extracellular metabolite measurements confirm our hypothesis for a spatiotemporally organized cross-feeding of pyruvate: cells at the swarm front consume malate, succinate and other potential preferred carbon sources, and secrete pyruvate. The secreted pyruvate is then utilized by cells that move over the same location in the agar at a later time, when malate and other preferred carbon sources are depleted. It is worth noting that the cells that secrete pyruvate are not the same cells that will consume pyruvate later; due to the movement of the swarm front, highly motile cells close to the front continue to move outwards as they leave pyruvate behind and the population of cells that feed on pyruvate are a mixture of cells that did not move with the swarm front and cells that migrated from more central regions of the swarm. The dynamic spatial structure of both the swarm community and its environment therefore plays a crucial role for the cross-feeding observed in this system, making it a truly spatiotemporal multicellular phenomenon, which is distinct from temporal pyruvate cross-feeding in liquid cultures^41–43^. A similar spatiotemporal cross-feeding interaction also occurs for fumarate (Extended Data Fig. 7). At the locations where pyruvate and fumarate are consumed, the spatiotemporal transcriptome data indicate that genes involved in the TCA cycle and respiration are upregulated (Supplementary Figs. 14 and 15), suggesting that the cells at these locations generate energy via oxidative phosphorylation.

## Discussion and Conclusions

Our analysis revealed all spatiotemporally regulated genes in *Bacillus subtilis* swarm development. We identified three major regions of the swarm with qualitatively different gene expression profiles and phenotypes: the region just behind the swarm front, the late swarm centre, and the intermediate spatiotemporal region between the front and the late swarm centre. At the swarm front, cells consume their preferred carbon sources, which are succinate and then malate in LB medium. At the swarm front, the cells also secrete pyruvate and fumarate, which are left in the agar across which the cells migrate. Our microscopy videos show that although the cells at the very edge of the swarm front are usually stuck or non-motile, the cells directly behind the leading edge are highly motile and frequently form rafts. Further inwards, motile cells coexist with non-motile elongated cells. In this intermediate region, biofilm matrix genes are beginning to be expressed, surfactin and fatty acids are synthesized, and the PBSX prophage is induced. In the late swarm centre, the preferred carbon sources have been depleted and cells consume pyruvate and fumarate, which were left behind in the agar by the preceding generations. As described previously^22,23^, cells in the late swarm centre form long threads that are present in multiple layers, resulting in a 3D biofilm. In previous work it was shown that these threads are cell chains^22^. Cell chain formation is associated with a low expression of the autolysin gene *lytF*^44^, which is consistent with the low expression levels of *lytF* we observed in the biofilm region (Supplementary Fig. 16). Between the gaps in the biofilm, a few short and motile cells are present. The three major regions of the swarm with distinct physiology and cellular behaviour display gradual transitions between each other and no sharp boundaries. We therefore do not expect that higher spatiotemporal resolution of transcriptomes would reveal further distinct regions of major consequence for the swarm development.

However, it is possible that phenotypic subpopulations that coexist within the same spatiotemporal locations^23,35^ are important for driving the swarm expansion. For example, in the late swarm centre, the long cell chains coexist with small motile cells, which are not distinguished by our transcriptome measurements. Furthermore, there are probably phenotypic differences of coexisting cells within the 3D biofilm due to resource gradients^6,45–47^. Similarly, in the intermediate region, long non-motile cells that sometimes form stationary clusters coexist with highly motile cells, yet again our transcriptome measurements do not distinguish these subpopulations. Potentially, these subpopulations can be distinguished by single-cell RNA-seq techniques that have emerged for bacteria, albeit with low genome coverage in their current versions^17,43,48,49^.

In summary, we reported simultaneous measurements of densely sampled spatiotemporal transcriptomes and microscopy-based biophysical properties in a developing microbial community. Comparing gene expression patterns and phenotype patterns, we observed a surprising disconnection between motility gene expression and cell speed, indicating the substantial influence of mechanisms beyond transcriptional regulation. By combining our spatiotemporal gene expression dataset with spatiotemporal metabolite measurements, we further discovered a spatiotemporally organized cross-feeding of pyruvate and fumarate, which are secreted by cells at the swarm front that perform substrate-level phosphorylation for energy generation. The secreted pyruvate and fumarate are left behind in the agar across which the cells migrate and are then consumed by a later generation of cells to run the TCA cycle. Due to the widespread conservation of the metabolic pathways involved in this spatiotemporal cross-feeding process, we expect this process to be ubiquitous for expanding microbial communities. More generally, the multilevel spatiotemporal datasets made available through this study provide the basis for the development of detailed spatiotemporal models that will connect gene expression, cellular phenotypes and biophysical dynamics within bacterial communities.

## Methods

### Swarming assay

All experiments were performed with a naturally competent derivative of the undomesticated *B. subtilis* NCIB 3610 strain, carrying the *comI*^Q12L^ allele^50^. Bacterial cultures for swarm inoculation were grown in Luria-Broth (LB) medium (Carl Roth, X968.3) for 16 h at 37 °C with shaking at 220 r.p.m. Swarming plates containing 9 ml of 0.5% Bacto agar (BD, 214010) in LB in 9 cm Petri dishes were dried for 10 min with an open lid at room temperature and then kept upside down at 37 °C for an additional 10–30 min before inoculation. Cells were transferred onto the swarming plate by dipping a sampling tip (see description of robotic sampling tips below) into the overnight culture and then lightly touching the surface of the swarming plate with this tip. This procedure resulted in a culture spot of diameter ~1 mm containing 3,000–5,000 cells on the agar surface.

After inoculation, the swarming plate was moved into a humidity chamber whose bottom was a microscope stage insert. This humidity chamber contained water-filled basins to ensure high humidity. The humidity chamber and large parts of the inverted microscope (Nikon Ti-E) were enclosed by a microscope incubator (Okolab). During the swarming assay, the temperature inside the Okolab enclosure was kept constant at 30 °C. The humidity chamber containing the agar plate and the stage insert was mounted on a motorized *xy* stage (Applied Scientific Instrumentation). Brightfield images were acquired using a ×16 water-immersion objective with numerical aperture 0.8 and an Andor Zyla 4.2PLUS sCMOS camera at 100 fps.

### Robotic sampling of cells for transcriptome measurements and imaging of cells during swarm development

We performed automated sampling of cells from the developing swarm using a custom-built robotic setup, which consisted of several motorized linear or rotational stages that collectively formed a robotic sampling arm, as schematically illustrated in Extended Data Fig. 1. All stages within the robotic sampling arm were controlled by the same Matlab algorithm that also controlled the motorized microscope *xy* stage, microscope camera and microscope focus to ensure that the robotic sampling arm is synchronized with the microscope movement and imaging.

One robotic sampling cycle consists of the following processes: Stages 1, 2 and 3.4 (stage numbers are defined in Extended Data Fig. 1) were translated such that a sampling tip was picked up from the tip box onto the tip holder attached to stage 3.4. Using rotational stage 3.3 and stage 1, the tip was then transported to the *xy* stage of the microscope where the swarming plate is located. The sampling tip was moved into the humidity-controlled incubation enclosure on the microscope *xy* stage (within which the swarming plate is located) through a hole in the cover of the enclosure, which can be opened and closed by stage 4. The sampling tip was then brought into the correct *xy* position for picking up cells using stages 1, 3.1 and 3.4, lowered onto the sample and brought in contact with the swarm surface by movement of stage 3.2, using live image analysis of the microscope camera’s field of view. After establishing contact with the swarm surface, the tip was kept at the surface for ~20 s before reversing the movement to retrieve the sampling tip. The duration for which the tip was in contact with the swarm surface was adapted according to the sampling position, with less contact time for dense regions. Control experiments using counting of colony forming units have shown that the sampling tip after this maneuver contains 10³–10^5^ cells. Finally, by moving stage 3.5, the tip was moved to the sample box and ejected into a 1.5 ml Eppendorf tube containing 50 µl of lysis buffer (40 U µl^−1^ Ready-lyse lysozyme (Lucigen, R1804M), 0.04 U µl^−1^ SUPERase in RNase Inhibitor (Thermo Fisher, AM2694), 10 mM Tris, adjusted to pH 8.0 with HCl (Thermo Fisher, 15568025), 1 mM EDTA (Thermo Fisher, AM9261)). The 50 µl of lysis buffer covered the cell-containing part of the tip. The Eppendorf tube was immediately manually collected, closed and snap-frozen in liquid nitrogen, followed by storage at −80 °C until RNA isolation. The Eppendorf tube containing the lysis buffer was kept on ice until a few minutes before being inserted into the sample ejection box on the robot. To maintain the low temperature of the lysis buffer during the time that it spends in the warm environment surrounding the microscope, Eppendorf tubes were kept in an ice-cold aluminum block, which was surrounded by isolation material. The aluminum block contains slots for up to six Eppendorf tubes and the aluminum block was exchanged every 5–8 min with an identical copy previously resting on ice. These steps to ensure a constant low temperature of the lysis buffer were necessary to retain the enzyme activity and minimize transcriptome changes and RNA degradation.

Manufacturing of the sampling tips for the robotic arm: We designed custom sampling tips to avoid scratches in the agar surface during the sampling procedure, which would alter the swarm development. These sampling tips consisted of a standard Eppendorf 10 µl pipette tip dipped into polydimethylsiloxane (PDMS, SYLGARD 184, Dow Corning) to produce a drop at the tip edge. To ensure reproducible PDMS drop sizes on the tip, custom holders for dipping 48 pipette tips simultaneously into a levelled bath of liquid PDMS were designed. The PDMS on the tips was then cured at 50 °C for 24 h. The cured sampling tips were then sterilized by dipping the tips into 70% ethanol, followed by drying at room temperature in the closed tip rack for 2 h. The sterilized sampling tips were used within 24 h.

Robotic sampling procedure in space and time: After the inoculation of the swarming plate as described in the section ‘Swarming assay’, growth of the cells was monitored by brightfield imaging until the end of the lag phase was reached. Once the swarm started expanding, robotic sampling was initiated. For the whole duration of the swarm expansion, until a final swarm diameter of 6 cm was reached, sets of spatially separated samples were acquired in 15–20-min intervals. For each set of spatially separated samples, a line was drawn from the centre of the swarm to the outer edge of the swarm, and samples were collected from up to 9 regularly spaced positions along this line (the exact number of sampled positions depends on the diameter of the swarm at any given time, and the spacing between sampling positions was at least 1 mm). The time for each sample to be acquired by the robot was ~30 s. To avoid sampling the same spot within the swarm too many times and therefore causing disruption to this area of the swarm, the innermost sample was chosen with a distance of 1.5 mm to the point of inoculation, and the angle between the *x* axis of the microscope stage and the line at which samples were taken was adapted by 30° after each run such that a sampling line with the same angle was only used for every 8th set of spatially separated samples. Using this procedure, samples were acquired from only half of the swarm area, whereas the other half of the swarm was untouched by the sampling tip. Immediately before and after sampling, an overview image of the sampling area was taken to document the impact of the sampling process on the swarm (Extended Data Fig. 4). These images were also used to estimate the number of cells extracted.

In addition to acquiring brightfield microscopy videos from the sampling location before sampling, we also acquired videos at spatial locations between the sampling locations. Furthermore, as control experiments, microscopy videos were acquired at corresponding spatial locations on the half of the swarm area that was untouched by the robotic sampling tip to determine whether the half of the swarm from which samples were acquired for transcriptome measurements developed differently from the half of the swarm that was untouched by the sampling tip.

### Sampling of cells during the lag phase for transcriptome measurements before swarm expansion

As the number of bacterial cells present on the agar surface during the lag phase is low and cell density plays an important role for the differentiation of cells during this period (for example, surfactin secretion, which is necessary to exit the lag phase, is controlled by quorum sensing), it was not possible to continuously acquire samples from the same swarm during the lag phase. Instead, several swarm plates were inoculated and incubated at the same time, and any given plate was only used for sampling once. To achieve a high reproducibility of lag phase development across the different plates, swarms were inoculated on single-well plates using the Echo 525 Acoustic liquid handler (Beckman Coulter, 001-10080) using an inoculation volume of 50 nl.

To collect cells from these plates, a small volume of lysis buffer previously kept on ice (3–4 µl) was pipetted onto the swarm surface and immediately retrieved by pipetting back up. This process of up-and-down pipetting was repeated 3 times to increase the number of collected cells. To increase the number of collected cells further, the process was repeated for several swarm colonies, and samples from the same timepoint were pooled in a final volume of 50 µl of lysis buffer, which was then immediately snap-frozen in liquid nitrogen, followed by storage at −80 °C until RNA isolation.

### RNA isolation and sequencing

Our experiments yielded two types of sample, which were snap-frozen in liquid nitrogen and then stored at −80 °C until RNA isolation. Sample type A: cells from the spatiotemporal sampling experiments during the swarm expansion phase, for which the cells are located on a sampling tip submerged in 50 µl of lysis buffer within an Eppendorf tube. Sample type B: cells from the temporal sampling experiments during the lag phase, for which the cells were directly collected in 50 µl of lysis buffer within an Eppendorf tube. In both cases, samples were thawed at room temperature, followed by incubation at room temperature for 5 min, interrupted every 1 min by vortexing to ensure complete lysis of cells. Then, the tip was removed and total RNA was extracted from this lysate using the hot SDS/hot phenol method^51^ with some modifications as follows. To the lysate, we added 6 µl of 1 M sodium acetate (pH 5.5, Sigma, S7899) and 62.5 µl of Roti-Aqua-Phenol (Carl Roth, A980) and incubated the mixture at 65 °C for 8 min. The whole mixture was transferred to a phase lock gel tube (VWR, 733-2478), followed by the addition of 62.5 µl chloroform (Sigma, C2432). The mixture was centrifuged at 21,130 × *g* for 15 min at 12 °C. The aqueous phase (~65–70 µl) was transferred to a new 0.2 ml PCR tube. RNA in the solution was purified by adding 120 µl of Agencourt RNAClean XP kit (Beckman Coulter, A63987). Samples were then treated with TURBO DNase (Thermo Fisher, AM2238) and the total RNA quality was analysed with a TapeStation 4150 (Agilent, G2992AA). According to the TapeStation results, several RNA samples with relatively high concentrations were diluted to ensure that a consistent amount of RNA was used for further processing to minimize sample-to-sample bias. For ribosomal RNA (rRNA) depletion in the total RNA, we used the ‘do-it-yourself’ method^52^ with reduced reaction volume, followed by purification of rRNA-depleted RNA using Agencourt RNAClean XP kit (Beckman Coulter, A63987). Sequencing library preparation was performed using NEBNext Ultra II Directional RNA Library Prep with sample purification beads (NEB, E7765S). Sequencing for swarming cells was carried out at the Max Planck Genome Centre (Cologne, Germany) using an Illumina HiSeq 3000 with 150 bp single reads (aiming for >5 M reads per library). Sequencing for lag-phase cells before swarm expansion was carried out at the Basel Genomics Facility (Basel, Switzerland) using an Illumina NovaSeq 6000 with 101-bp single reads (aiming for >5 M reads per library).

### Extracellular metabolite measurements during swarm development

To measure extracellular metabolites in the agar underneath the cells, swarms were allowed to develop to the desired diameter. Then, cells were removed from the agar by gently scratching the surface with a razor blade. At specific radii, a biopsy of the agar was acquired (the full depth of the agar) to acquire ~20 mg of agar, which was placed in an Eppendorf tube. Samples were weighed using a fine scale, and the appropriate amount of metabolite extraction solution (10 µl mg^−1^) containing 50% TE buffer (10 mM Tris, adjusted to pH 7.0 with HCl (Thermo Fisher, AM9850G), 1 mM EDTA (Thermo Fisher, AM9261)) and 50% methanol was added. Eppendorf tubes were then kept shaking at 4 °C for 2 h and afterwards centrifuged at 4 °C for 10 min at 21,130 × *g*. The liquid phase was then filtered using a 0.22 µm filter (regenerated cellulose, 4 mm diameter, Phenomenex, AF0-3203-52) and stored at −20 °C until further processing by mass spectrometry. In addition to the dilution of the samples by 1:10 resulting from adding the extraction solution, the extracts were diluted further by 1:20 for amino acid measurements to optimize the detection by the instruments. For organic acids, the extracts were used without further dilution.

#### Amino acids

Quantitative determination of amino acids was performed using HRES LC–MS. The chromatographic separation was performed on an Agilent Infinity II 1260 HPLC system using a ZicHILIC SeQuant column (150 × 2.1 mm, 5 μm particle size, 100 Å pore size) connected to a ZicHILIC guard column (20 × 2.1 mm, 5 μm particle size) (Merck KgAA). We used a constant flow rate of 0.3 ml min^−1^, with mobile phase A being 0.1% formic acid in a 99:1 mixture of water:acetonitrile (Honeywell) and phase B being 0.1% formic acid in a 99:1 mixture of acetonitrile:water (Honeywell) at 25 °C. The injection volume was 1 µl. The profile of the mobile phase consisted of the following steps and linear gradients: 0–8 min from 80% to 60% B; 8–10 min from 60% to 10% B; 10–12 min constant at 10% B; 12–12.1 min from 10% to 80% B; 12.1–14 min constant at 80% B. An Agilent 6470A mass spectrometer was used in positive mode with an electrospray ionization source and the following conditions: ESI spray voltage 4,500 V, nozzle voltage 1,500 V, sheath gas 400 °C at 12 l min^−1^, nebulizer pressure 30 psig and drying gas 250 °C at 11 l min^−1^. Compounds were identified on the basis of their mass transition and retention time compared to standards. Chromatograms were integrated using MassHunter software (Agilent). Absolute concentrations were calculated on the basis of an external calibration curve prepared in the sample matrix. To mimic the sample matrix, an aliquot of freshly prepared agar was treated similar to the extraction performed on samples for exometabolome determination. Mass transitions, collision energies, cell accelerator voltages and dwell times were optimized using chemically pure standards. Parameter settings of all targets are given in Supplementary Table 1.

#### Organic acids

Quantitative determination of organic acids was performed using LC–MS/MS. The chromatographic separation was performed on an Agilent Infinity II 1290 HPLC system using a Kinetex EVO C18 column (150 × 2.1 mm, 3 μm particle size, 100 Å pore size, Phenomenex) connected to a guard column of similar specificity (20 × 2.1 mm, 3 μm particle size, Phenomoenex). We used a constant flow rate of 0.2 ml min^−1^, with mobile phase A being 0.1% formic acid in water and phase B being 0.1% formic acid in methanol (Honeywell) at 25 °C. The injection volume was 0.5 µl. The profile of the mobile phase consisted of the following steps and linear gradients: 0–2.5 min constant at 0% B; 2.5–6 min from 0% to 100% B; 6–8 min constant at 100% B; 8–8.1 min from 100% to 0% B; 8.1–12 min constant at 0% B. An Agilent 6495 ion funnel mass spectrometer was used in negative mode with an electrospray ionization source and the following conditions: ESI spray voltage 2,000 V, nozzle voltage 500 V, sheath gas 260 °C at 10 l min^−1^, nebulizer pressure 35 psig and drying gas 100 °C at 13 l min^−1^. Compounds were identified on the basis of their mass transition and retention time compared to standards. Chromatograms were integrated using MassHunter software (Agilent). Absolute concentrations were calculated on the basis of an external calibration curve prepared in the sample matrix. To mimic the sample matrix, an aliquot of freshly prepared agar was treated similar to the extraction performed for exometabolome determination. Mass transitions, collision energies, cell accelerator voltages and dwell times were optimized using chemically pure standards. Parameter settings of all targets are given in Supplementary Table 1.

### Transcriptome data analysis

For each of the FASTQ files from the 284 samples of the swarm expansion phase (3 replicates: replicate 1 contained 96 samples, replicate 2 contained 92 samples, replicate 3 contained 96 samples), we performed read trimming using Trimmomatic (v.0.39)^53^ and mapped the trimmed reads to the *B. subtilis* NCIB 3610 reference genome and plasmid (NCBI accession number: NZ_CP020102 and NZ_CP020103) using HISAT2 (v.2.2.1)^54^ in single-end mode. Reads mapped to all functional genes were counted using featureCounts (v.2.0.1)^55^ with fractional counting for multimapping and multi-overlapping reads (−OM−−fraction) in a strand-specific manner (−s 2). Any short transcripts such as small RNA or transfer RNA should not be detected because the above-described RNA purification steps using RNAClean XP kit can only retain transcripts bigger than 200 nt. Reads that were mapped to protein-coding sequences, non-coding RNAs, transfer-messenger RNA, signal recognition particle RNA and ribonuclease P RNA (in total 4,342 genes) were used for downstream analyses.

Raw read counts of the expansion phase samples were loaded into R and filtered by keeping all genes for which there were at least 2 samples with a read count of at least 10, leaving 3,932 genes out of 4,342 genes. After further removing samples with total read counts of <10^6^ mapped to those genes, there were 278 samples left: 95 samples in replicate 1, 92 samples in replicate 2, and 91 samples in replicate 3, which were analysed further. We then generated a DGEList object containing all 278 transcriptomes and applied the TMM normalization method implemented in edgeR (v.3.26.8)^56,57^ for samples pooled from all three experiments, using the calcNormFactors function to enable sample-to-sample comparison of the data. All subsequent analysis was then performed with normalized log_2_ values.

For Supplementary Fig. 21, we performed the same processing for the FASTQ files of the 33 lag phase samples and then performed the normalization using both the 278 expansion phase and 33 lag phase samples, following the same criteria. This resulted in a final dataset comprising 4,083 genes across 311 samples. The normalization in Supplementary Fig. 21 therefore differs from that used for all other figures.

### Spectral representation of spatiotemporal gene expression

The transcriptome measurements and the microscopy-based measurement of phenotypic properties were sampled at a set of radial space–time points $\{{{\bf{r}}}_{l}{\boldsymbol{=}}\left({t}_{l},{p}_{l}\right){\}}_{l=1}^{L}$, where ${{\rm{t}}}_{l}$ is the time and ${p}_{l}$ is the radial position from the centre of the swarm at which the sample was acquired. For each gene, we have a sample vector ${{\bf{g}}}_{n}$ with length $L$, where the $l$ th entry is the gene expression at the point ${{\bf{r}}}_{{\boldsymbol{l}}}$. Similarly, for each phenotypic property, we have a sample vector ${{\mathbf{\Phi }}}_{n}$ with length $L$.

**Extended Data Fig. 9:**
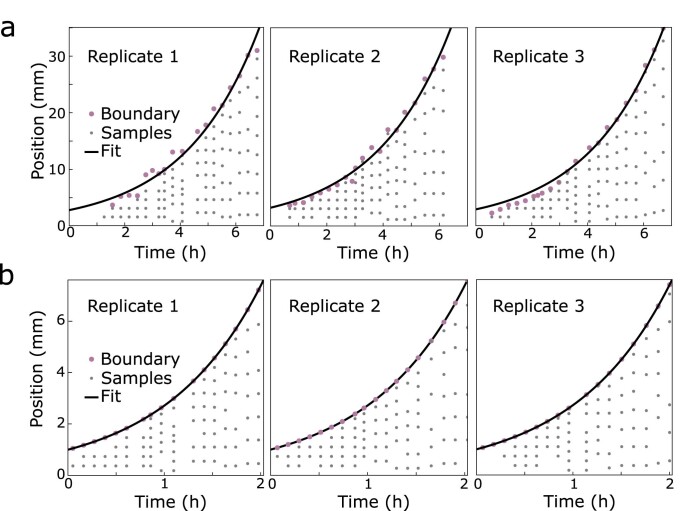
An exponential fit approximates the boundary of swarm expansion and can be used to define a common spatiotemporal domain for all replicates. **(a)**, An exponential function is fitted to the space-time location of boundary points (indicated in purple colour) for each data set. Space-time coordinates are then rescaled as described in the *Methods* section, and the rescaled space-time coordinates are shown in their common non-dimensionalized domain in panel **(b)**.

To form a spectral representation across the three replicates, we fit a common domain to the three replicates. Experimentally, the radial position of the boundary ${b}_{l}$ of the swarm at each time ${t}_{l}$ was determined automatically by detecting the presence and location of bacteria in the microscopy field of view and moving the microscope stage until the field of view was split between colonized agar containing bacteria and uncolonized agar in approximately equal proportions. We simultaneously fitted a boundary of the form $b\left(t\right)={b}_{0}\exp \left(t/\tau \right)$ to all replicates by first minimizing the loss function,

1 $${\rm{L}}\left({{\tau }},{b}_{0}^{\left(1\right)},{b}_{0}^{\left(2\right)},{b}_{0}^{\left(3\right)}\right)=\mathop{\sum }\limits_{k=1}^{3}\mathop{\sum }\limits_{l=1}^{{L}^{\left(k\right)}}{\left({b}_{i}^{\left(k\right)}-{b}_{0}^{\left(k\right)}\exp \left({t}_{l}^{\left(k\right)}/{{\tau }}\right)\right)}^{2}$$

where superscript ^(*n*)^ denotes the index of the three different replicates. This exponential fit approximates the experimental data very well (Extended Data Fig. 9a).

To obtain non-dimensional data, we rescaled data as follows. Let $r$ be the index corresponding to the largest ${b}_{0}^{\left(k\right)}$; we defined the time shift ${t}_{s}^{\left(r\right)}={t}_{0}^{\left(r\right)}$ and scaled the initial value ${b}_{0}={b}_{0}^{\left(r\right)}\exp \left({t}_{s}^{\left(r\right)}/\tau \right)$. The other time shifts are given by

2 $${t}_{s}^{\left(k\right)}={\rm{\tau }}\left[\log \left({b}_{0}^{\left(r\right)}\right)-\log \left({b}_{0}^{\left(k\right)}\right)\right]+{t}_{s}^{\left(r\right)}.$$

We then non-dimensionalized the data ($\widetilde{\bullet }$ variables) using ${\widetilde{t}}_{l}=\left({{\rm{t}}}_{l}-{t}_{s}^{\left(k\right)}\right)/{\rm{\tau }}$ and ${\widetilde{p}}_{l}={p}_{l}/{{\rm{b}}}_{0}$. The domain boundary is then given by $0\le \widetilde{t}\le T$ and $0\le \widetilde{r}\le \exp \left(\widetilde{t}\right)$ where $T$ is maximum non-dimensional time present in all three replicates. Data points that lay outside the domain after rescaling were not used in the spectral representation. The non-dimensionalized domain and how the individual sampling data points are distributed within this domain are shown in Extended Data Fig. 9b.

We built a domain-specific orthogonal polynomial basis ${{{\rm{P}}}_{m}\left(\widetilde{t},\widetilde{p}\right)}_{m=0}^{M}$ by applying Gram–Schmidt orthogonalization^58^ to the monomial set,

3 $$\{1,\widetilde{t},\widetilde{p},{\widetilde{t}}^{2},\widetilde{t}\widetilde{p},{\widetilde{p}}^{2},\ldots \},$$

under the inner product

4 $$\left\langle f,g\right\rangle ={\int }_{0}^{{T}}\text{d}\widetilde{t}{\int }^{{\exp \left(\widetilde{t}\right)}}_{0}\text{d}\widetilde{r}{e}^{-\widetilde{t}}{fg}.$$

The space–time dependence of each gene was compressed by expanding each sample vector,

5 $${{\bf{g}}}_{n}=\mathop{\sum }\limits_{m=0}^{{\rm{M}}}{c}_{m,n}{{\bf{P}}}_{{\rm{m}}}$$

where ${{\bf{P}}}_{{\rm{m}}}$ is the length ${\rm{L}}$ vector formed by evaluating ${{\rm{P}}}_{{\rm{m}}}\left(\widetilde{t},\widetilde{p}\right)$ at each non-dimensional space–time point $({\widetilde{t}}_{l},{\widetilde{p}}_{l})$. The coefficients were fitted using least squares on the matrix equation,

6 $${{\bf{g}}}_{n}=\left[\begin{array}{cccc}{{\bf{P}}}_{0} & {{\bf{P}}}_{1} & \cdots & {{\bf{P}}}_{M}\end{array}\right]\left[\begin{array}{c}{{\rm{c}}}_{0,{\rm{n}}}\\ {{\rm{c}}}_{1,{\rm{n}}}\\ \vdots \\ {{\rm{c}}}_{{\rm{M}},{\rm{n}}}\end{array}\right]$$

for each gene and property.

To combine information from all replicates, we fitted a single coefficient vector for the replicates by stacking the least square problems on top of each other to form a single linear regression problem,

7 $$\left[\begin{array}{c}{{\bf{g}}}_{n}^{{\boldsymbol{(}}{\bf{1}}{\boldsymbol{)}}}\\ {{\bf{g}}}_{n}^{{\boldsymbol{(}}{\bf{2}}{\boldsymbol{)}}}\\ {{\bf{g}}}_{n}^{{\boldsymbol{(}}{\bf{3}}{\boldsymbol{)}}}\end{array}\right]=\left[\begin{array}{c}\begin{array}{cccc}{{\bf{P}}}_{0}^{(1)} & {{\bf{P}}}_{1}^{(1)} & \cdots & {{\bf{P}}}_{M}^{(1)}\end{array}\\ \begin{array}{cccc}{{\bf{P}}}_{0}^{(2)} & {{\bf{P}}}_{1}^{(2)} & \cdots & {{\bf{P}}}_{M}^{(2)}\end{array}\\ \begin{array}{cccc}{{\bf{P}}}_{0}^{(3)} & {{\bf{P}}}_{1}^{(3)} & \cdots & {{\bf{P}}}_{M}^{(3)}\end{array}\end{array}\right]\left[\begin{array}{c}{\bar{{\rm{c}}}}_{0,{\rm{n}}}\\ {\bar{{\rm{c}}}}_{1,{\rm{n}}}\\ \vdots \\ {\bar{{\rm{c}}}}_{{\rm{M}},{\rm{n}}}\end{array}\right],$$

from which a smooth average spatiotemporal gene expression was formed,

8 $${\bar{{\rm{g}}}}_{n}(\widetilde{t},\widetilde{p})=\mathop{\sum }\limits_{m=0}^{{\rm{M}}}{\bar{c}}_{m,n}{{\rm{P}}}_{{\rm{m}}}(\widetilde{t},\widetilde{p}).$$

The same procedure was also used to produce average properties ${\bar{\Phi }}_{n}$.

### Spatiotemporal pattern identification

The coefficients ${c}_{m,n}$ for the basis functions ${\bf{P}}_{{\rm{m}}}$ encode information about the spatiotemporal expression pattern of the genes and phenotypic properties, which allowed us to cluster genes on the basis of their spatiotemporal expression pattern using the spectral coefficients ${c}_{m,n}$ directly. With the clustering analysis, we intended to identify the underlying patterns independent of global shifts and scaling. Therefore, we defined coefficients with the mean subtracted and scaled by the standard deviation,

9 $${k}_{m,n}=\frac{{{c}_{m,n}-{\delta }_{n,0}\mu }_{n}/{p}_{0}}{{\sigma }_{n}}$$

where ${\sigma }_{n}$ is the standard deviation of the expression of gene $n$, ${\mu }_{n}$ is the mean of the expression of gene $n$, ${\delta }_{n,m}$ is the Kronecker delta that is 0 when $n\ne m$ and 1 when $n=m$, and ${p}_{0}$ is constant since it is a degree 0 polynomial. Note that under this rescaling, ${k}_{m,0}$ is no longer an independent parameter since it is fully determined by the means of the higher-order polynomials and their respective coefficients. Using these rescaled coefficients ${k}_{m,n}$, we defined a score for how strongly patterned a gene expression profile is. A spatiotemporally patterned expression profile should have two components:

The pattern should vary smoothly so that the pattern should be well approximated by the spectral representation, which means that the scaled representation error

10 $${{\mathscr{E}}}_{{\mathcal{n}}}={\left\Vert\frac{{{\bf{g}}}_{{\boldsymbol{n}}}-{\mu }_{n}}{{\sigma }_{n}}-\mathop{\sum }\limits_{m=0}^{M}{k}_{m,n}{{\bf{P}}}_{{\bf{m}}}\right\Vert}^{2}$$

should be small.

The pattern should not just be constant, meaning that the higher coefficients should be important, which also means that the pattern score

11 $${{\mathscr{P}}}_{{\mathcal{n}}}=\mathop{\sum }\limits_{m=1}^{M}{k}_{m,n}^{2}$$

should be large.

We therefore defined a space–time ranking,

12 $${{\mathscr{R}}}_{{\mathcal{n}}}=\frac{{{\mathscr{P}}}_{{\mathcal{n}}}}{{{\mathscr{E}}}_{{\mathcal{n}}}}$$

which is large when a gene expression has a strong spatiotemporal pattern, and low when there is no spatiotemporal pattern. For identifying different types of spatiotemporal pattern on the basis of the spectral coefficients ${c}_{m,n}$, we kept only those genes with a high ranking ${{\mathscr{R}}}_{{\mathcal{n}}}$. We defined the cut-off for which genes were designated as displaying a spatiotemporal pattern by ordering the genes by their ranking (smallest to largest) and then finding the largest integer ${N}_{c}$ such that,

13 $$\frac{\mathop{\sum }\nolimits_{n=1}^{{N}_{c}}{\mathscr{R}}_{{\mathcal{n}}}}{\mathop{\sum }\nolimits_{n=1}^{N}{\mathscr{R}}_{{\mathcal{n}}}}\le 0.5.$$

For the genes that displayed a spatiotemporal pattern on the basis of the above criterion, we calculated the cosine similarity between the coefficients,

14 $${d}_{{np}}=\frac{\mathop{\sum }\nolimits_{m=1}^{M}{k}_{m,n}{k}_{m,p}}{\sqrt{\mathop{\sum }\limits_{m=1}^{M}{k}_{m,n}^{2}}\sqrt{\mathop{\sum }\limits_{m=1}^{M}{k}_{m,p}^{2}}}.$$

**Extended Data Fig. 10:**
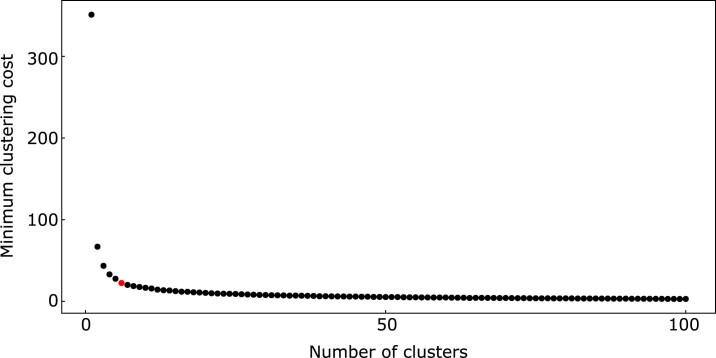
Minimum clustering cost as a function of cluster number, used for the identification of the number of different spatiotemporal gene expression patterns. With an increasing number of clusters, the minimum clustering cost decreases, with an initially sharp decline that levels off for high cluster numbers. Highlighted in red is the location of 6 clusters, the number which was chosen for further analyses in Fig. 2.

We used cosine distances since we needed a metric that is independent of a global scaling to the expression level. The distance matrix $D=\left({d}_{{np}}\right)$ was then separated into clusters using the *k*-medoids algorithm^59^ to produce *k* distinct patterns. The choice of the number of clusters was based on a plot of the total cost versus number of clusters and choosing a value in the elbow of the curve (Extended Data Fig. 10). From each cluster, we chose the highest ranked gene as the most representative spatiotemporal pattern.

### Image analysis

To perform single-cell segmentation on the brightfield microscopy images of the swarm, we used Stardist^60^. Bacterial movement fields were calculated using the Horn–Schunck optical flow method^61^, applied to consecutive images of a video. From the segmentation and bacterial movement fields, all phenotypic properties listed in Supplementary Table 2 were calculated. For properties calculated for each individual cell, median values were used across one image field of view, and mean values across all 48 frames in a video were used for visualization in heat maps. The following paragraphs describe the definitions of emergent property parameters.

#### Nematic order

The nematic order parameter $S\left(\vartheta \right)$ for two cells with a relative angle $\vartheta$ was quantified as

15 $$S\left(\vartheta \right)=1.5\cdot { \cos \left(\vartheta \right)}^{2}-0.5.$$

The local nematic order parameter for a specific cell in a swarm was then defined as the mean of the nematic order parameter of the cell with each of its neighbours within a centroid–centroid distance of ≤10 µm.

#### Non-motile clusters

Non-motile cells were identified by thresholding of the bacterial movement field with a cut-off of 8 µm s^−1^ and by hierarchical clustering based on centroid–centroid distance with a cut-off of 2 µm. Only clusters with 10 or more cells were considered.

#### Rafts

The neighbourhood of each cell was defined as all cells within a centroid–centroid distance of 30 µm to the cell under investigation. To quantify rafting behaviour, we counted the number of motile cells (speed of 10 µm s^−1^ or more) in this neighbourhood that share the same orientation as the cell under investigation up to a tolerance of 15°. This number was then divided by the total number of cells in the neighbourhood. This ratio, called the ‘local rafting ratio’, was calculated for each cell and used as a measure for local rafting activity.

#### Density fluctuations

To calculate density fluctuations, the image was split into sub-images of size 120 × 120 pixels (~48 × 48 µm^2^) and for each sub-image, the density was calculated as the fraction of the area covered by cells to the entire area. For the density fluctuations in space, the standard deviation between the local densities in sub-images was calculated for each timepoint. The final value was taken to be the mean across all timepoints.

### Reporting summary

Further information on research design is available in the Nature Portfolio Reporting Summary linked to this article.

### Supplementary information

Supplementary Information Supplementary Tables 1–3 and Figs. 1–21.

Reporting Summary

Supplementary Video 1 Top view of the swarm expansion process, showing three replicates. The swarm appears as a slightly brighter area on the agar plate and uniformly expands outwards.

Supplementary Video 2 Movie of the sampling process using the robotic setup. Shown is one cycle of sampling, during which the robot picks up a sampling tip, moves it to the swarming plate and brings it into contact with the swarm. It then moves backwards and ejects the tip into an Eppendorf tube.

## Data Availability

Transcriptome spatiotemporal heat maps and heat maps of microscopy-based measurements are available in a curated dataset explorer at https://drescherlab.org/data/swarm-transcriptome/. Transcriptome data that were used to generate the heat maps are also available at the National Center for Biotechnology Information Gene Expression Omnibus under the accession number GSE224332 (https://www.ncbi.nlm.nih.gov/geo/query/acc.cgi?acc=GSE224332). Files for viewing a 3D CAD model of the robotic sampling system are available on Zenodo (10.5281/zenodo.8229225) and can be viewed with the AutoDesk Inventor software. Coefficients of the spectral representation of each gene and image-analysis-based physical property as well as pattern identification and multidimensional scaling coordinates used in Fig. 2 are available on Zenodo (10.5281/zenodo.8355669). Metabolomics data are also available on Zenodo (10.5281/zenodo.8348257). Raw imaging data (1.9 terabyte in size) are available from the corresponding authors upon reasonable request, if infrastructure for transferring and storing these data are available.

## Code availability

Source code of the Matlab scripts used to measure swarm properties by image analysis and R scripts used to normalize transcriptome data are available in a GitHub repository at https://github.com/knutdrescher/swarm-transcriptome.
