# Supplementary material for: Simultaneous spatiotemporal transcriptomics and microscopy of Bacillus subtilis swarm development reveal cooperation across generations
Source: Nat Microbiol. 2023 Nov 16;8(12):2378–91. doi: 10.1038/s41564-023-01518-4 (PMC10686836; doi:10.1038/s41564-023-01518-4)
Supplement: Supplementary file 1 — Supplementary Tables 1–3 and Figs. 1–21. [file 41564_2023_1518_MOESM1_ESM.pdf]

nature microbiology

Article

https://doi.org/10.1038/s41564-023-01518-4

Simultaneous spatiotemporal transcriptomics and microscopy of *Bacillus subtilis* swarm development reveal cooperation across generations

In the format provided by the authors and unedited

Table of Contents

- Tables S1-S3
- Figures S1-S21
- References in the Supplementary Information

Supplementary Tables

Table S1: Measurement parameter settings for mass spectrometry based detection of amino acids and organic acids: Mass transitions, collision energies, cell accelerator voltages and dwell times have been optimized using chemically pure standards.

Name	Precursor ion m/z (Da/eV)	Product ion m/z (Da/eV)	Dwell (10^{-3} s)	Fragmentor voltage (V)	Collision energy (A.U.)	Cell accelerator (A.U.)	Polarity
Tryptophane	205	188	20	90	7	5	Positive
Tryptophane	205	146	20	90	17	5	Positive
Tyrosine	182.1	165	20	100	6	5	Positive
Tyrosine	182.1	136	20	100	12	5	Positive
Arginine	175	116	20	100	12	5	Positive
Arginine	175	70.2	20	100	28	5	Positive
Phenlalanine	166	120.1	20	90	14	5	Positive
Phenlalanine	166	103.1	20	90	30	5	Positive
Histidine	156	110.1	20	120	14	5	Positive
Histidine	156	83.1	20	120	28	5	Positive
Methionine	150	133	20	90	7	5	Positive
Methionine	150	104	20	90	7	5	Positive
Glutamate	148.1	84.1	20	80	17	5	Positive
Glutamate	148.1	56.1	20	80	34	5	Positive
Lysine	147.1	130.1	20	80	7	5	Positive
Lysine	147.1	84.1	20	80	16	5	Positive
Glutamine	147	130	20	80	7	5	Positive
Glutamine	147	84.1	20	80	17	5	Positive
Aspartate	134.1	88	20	80	9	5	Positive
Aspartate	134.1	74	20	80	14	5	Positive
Asparagine	133.1	87.1	20	80	17	5	Positive
Asparagine	133.1	74	20	80	16	5	Positive
Leucine	132.1	86.2	20	90	7	5	Positive
Isoleucine	132.1	86.2	20	90	8	5	Positive
Isoleucine	132.1	69.1	20	90	18	5	Positive
Leucine	132.1	30.3	20	90	18	5	Positive
Cystein	122	76.1	20	80	13	5	Positive
Cystein	122	59.2	20	80	24	5	Positive
Threonin	120.2	74.1	20	90	8	5	Positive
Threonin	120.2	55.9	20	90	18	5	Positive
Valine	118.1	72	20	90	9	5	Positive
Valine	118.1	55.1	20	90	23	5	Positive
Proline	116	70.2	20	90	16	5	Positive
Proline	116	43.3	20	90	35	5	Positive
Serine	106.1	60.2	20	90	12	5	Positive
Serine	106	50.1	20	90	7	5	Positive
Serine	106	41.2	20	90	11	5	Positive
Alanin	90	44.1	20	80	12	5	Positive
Citrate	191	111.1	20	380	11	5	Negative
Citrate	191	85.1	20	380	14	5	Negative
Malate	133.1	115.1	20	380	8	5	Negative
Malate	133.1	71.2	20	380	12	5	Negative
Succinate	117.2	73.2	20	380	9	5	Negative

Succinate	117.2	55.1	20	380	15	5	Negative
Fumarate	115.1	71.2	20	380	4	5	Negative
Fumarate	115.1	27.3	20	380	9	5	Negative
Lactate	89.2	89.2	20	380	0	5	Negative
Lactate	89.2	71.3	20	380	10	5	Negative
Pyruvate	87.1	87.1	20	380	0	5	Negative
Pyruvate	87.1	43.1	20	380	4	5	Negative
Glycolate	75.2	75.2	20	380	0	5	Negative
Glycolate	75.2	47.2	20	380	6	5	Negative
Glyoxylate	73.2	73.2	20	380	0	5	Negative
Glyoxylate	73.2	45.2	20	380	7	5	Negative

Table S2. List and short description of properties determined by image analysis.

Property name	Description	Units
**Local Properties**		
Cell speed	Speed of an individual cell	µm/s
Specific kinetic energy	Square of the cell speed	$\mu m^2/s^2$
Cell area	Number of pixels belonging to a cell according to the segmentation, converted to μm^2	μm^2
Cell aspect ratio	Major Axis length divided by minor axis length	No unit
Nematic order (orientation)	Nematic order parameter based on cell´s orientation of neighbours within a range of 10 µm	No unit
Local biomass density	Area fraction within a circle around the cell´s centroid of radius 5 µm	Fraction
Local number density	Number of neighbours whose centroid-centroid distance to cell of interest is smaller than 10 µm, divided by the area of a circle of radius 10 µm	$\#/\mu m^2$
Local rafting factor	Number of motile cell within neighbourhood of a cell that share this cell´s orientation up to tolerance of 15°	No unit
**Global properties**		
Global biomass density	Area fraction covered by cells in the field of view	Fraction
Global number density	Number of cells in the field of view, divided by field of view size	$\#/\mu m^2$
Biomass density fluctuations in space	Standard deviation between local densities measured in sub-images of size 48x48µm	No unit
Fraction of non-motile cells	Number of non-motile cells divided by total number of cells in the field of view	Fraction
Fraction of rafting cells	Number of rafting cells divided by total number of cells in the field of view	Fraction
Number of non-motile clusters	Number of non-motile clusters in the field of view divided by field of view size	$\#/\mu m^2$
Size of non-motile clusters	Median number of cells per non-motile cluster	# cells

Table S3: List of commercial components used for constructing the sampling robot.

Description of component	Product number	Vendor	Amount
Stepper motor lid samples	154-22-386	Distrelec	1
Limit switch	682-2626	RS Components	4
Stepper motor lid tip box	EXP-R25-073	Exp-tech	1
Arduino motor shield	EXP-R15-151	Exp-tech	1
Arduino	EXP-R08-002	Exp-tech	1
Power supply for arduino	EXP-T05-004	Exp-tech	1
Stage 1	SLLV42-400-LC/1-L	Smaract	1
Stage 2	SLLV42-400-LC/1-L	Smaract	1
Stage 3.1	SLS-5252-D-LC	Smaract	1
Stage 3.2	SLS-5252-D-LC	Smaract	1
Stage 3.3	SR-5014 -D-L	Smaract	1
Stage 3.4	SLC-1780-D-LC	Smaract	1
Stage 3.5	SLC-1730-D-LC	Smaract	1
Stage 4	SLC-1770-LC	Smaract	1
Angle bracket	SBB:C-0005	Smaract	1
Constant force spring	SBB:T-0001	Smaract	1
Tools	SBB:T-0001	Smaract	1
Controller	MCS2-C-0008	Smaract	1
Sensor module	MCS2-S-0001	Smaract	3
Manual controller	MCS2-H-0001	Smaract	1

Supplementary Figures

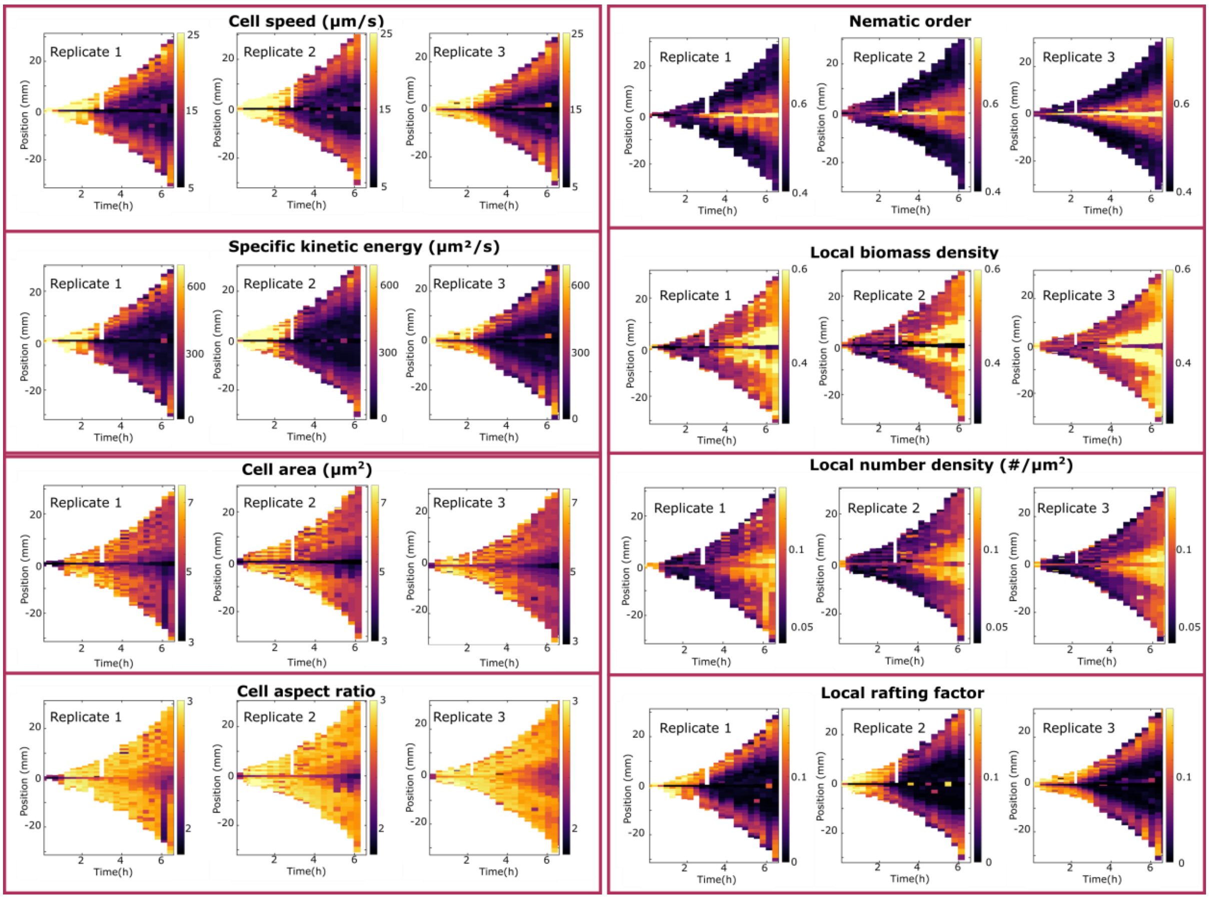

Figure S1: Spatiotemporal measurements of local properties during swarm development. Based on microscopy measurements and images analysis, measurements of the local single cell properties cell speed, cell area, and cell aspect ratio, as well as the emergent single-cell property nematic order are shown in spatiotemporal heatmaps for all three experimental replicates. Results are consistent across replicates and there is no significant difference between the sampled region of the swarm (top half, position > 0) and the unsampled region (bottom half, position < 0).

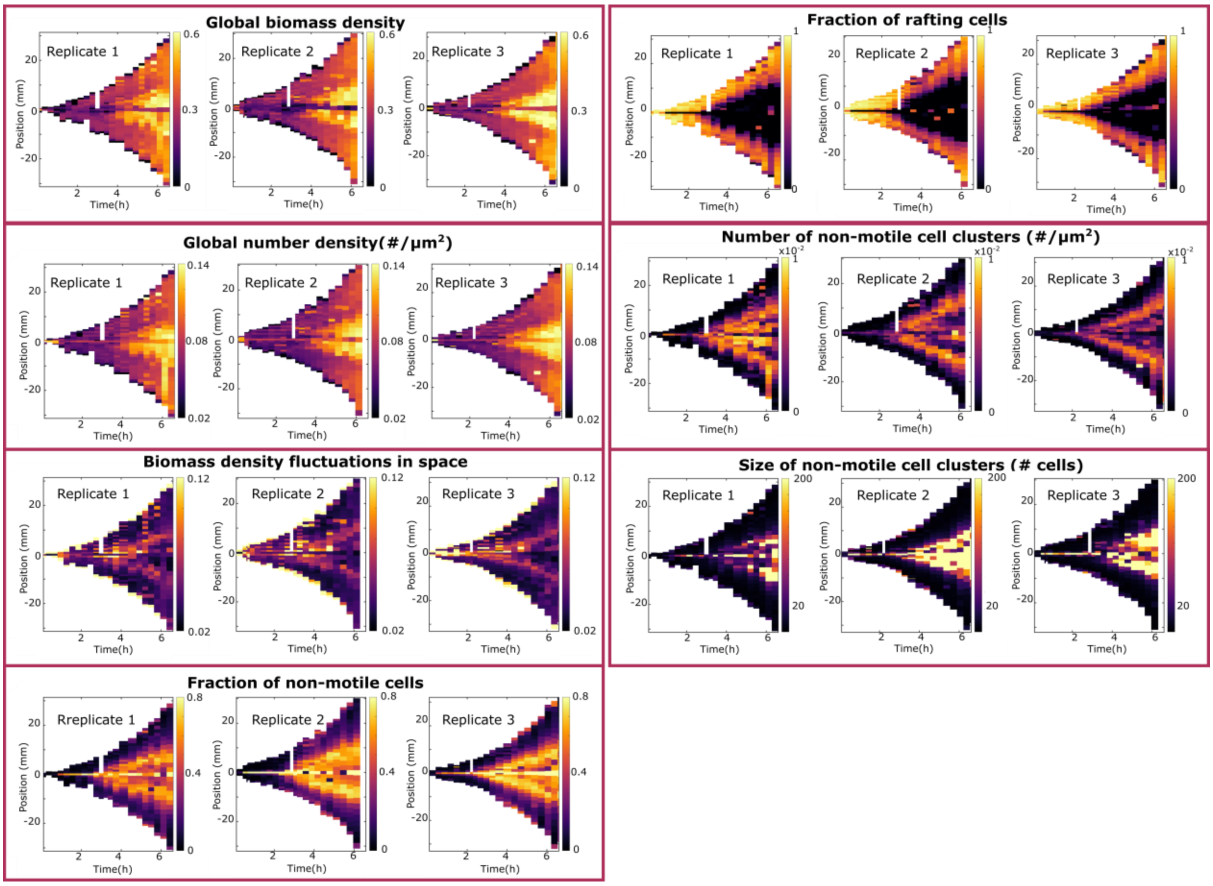

Figure S2: Spatiotemporal measurements of global properties during swarm development. Based on microscopy measurements and images analysis, measurements of the global biomass density, global number density, biomass density fluctuations in space, and the fraction of non-motile cells are shown in spatiotemporal heatmaps for all three experimental replicates. “Global” parameters are those that are measured for one microscopy field of view without spatial resolution inside the field of view. Results are consistent across replicates and there is no significant difference between the sampled region of the swarm (top half, position > 0) and the unsampled region (bottom half, position < 0).

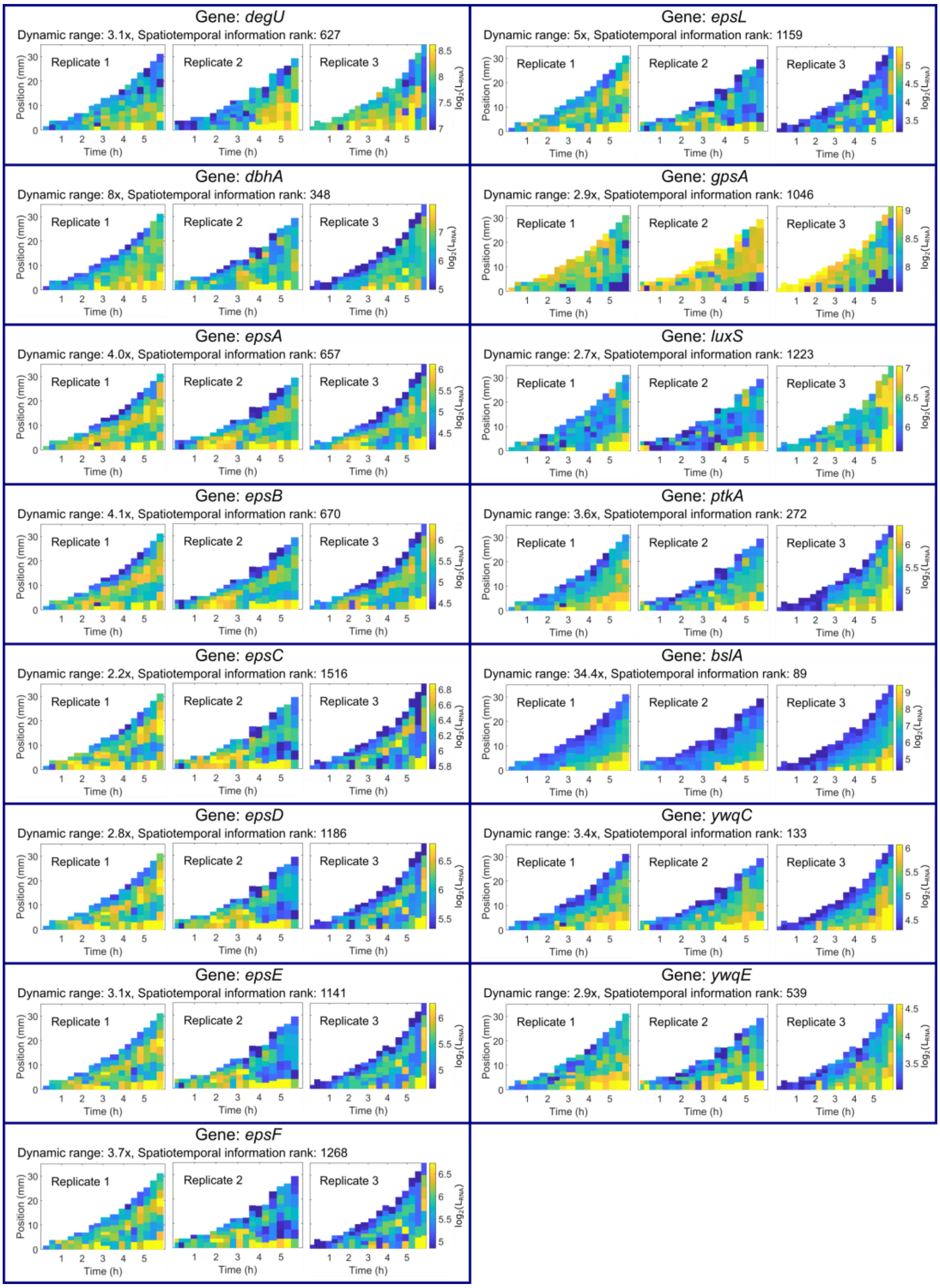

Figure S3: Biofilm genes and their spatiotemporal expression. Spatiotemporal heatmaps show the gene expression during swarm development, and the colour of each tile in a heatmap indicates the expression level L_{RNA} of a particular gene. The dynamic range of a gene is defined as the ratio between the highest and the lowest colour bar value, which are the 95th and 5th percentile of gene expression values of all samples, respectively. The spatiotemporal information rank of a gene is introduced in Fig. 2b, where genes are ranked according to their spatiotemporal information score, with a higher rank value corresponding to lower spatiotemporal information. Shown are the spatiotemporal gene expression heatmaps of genes related to biofilm matrix production and biofilm regulation. Many genes are upregulated at the late swarm centre, but the *eps* genes are upregulated both at the swarm centre and the intermediate region.

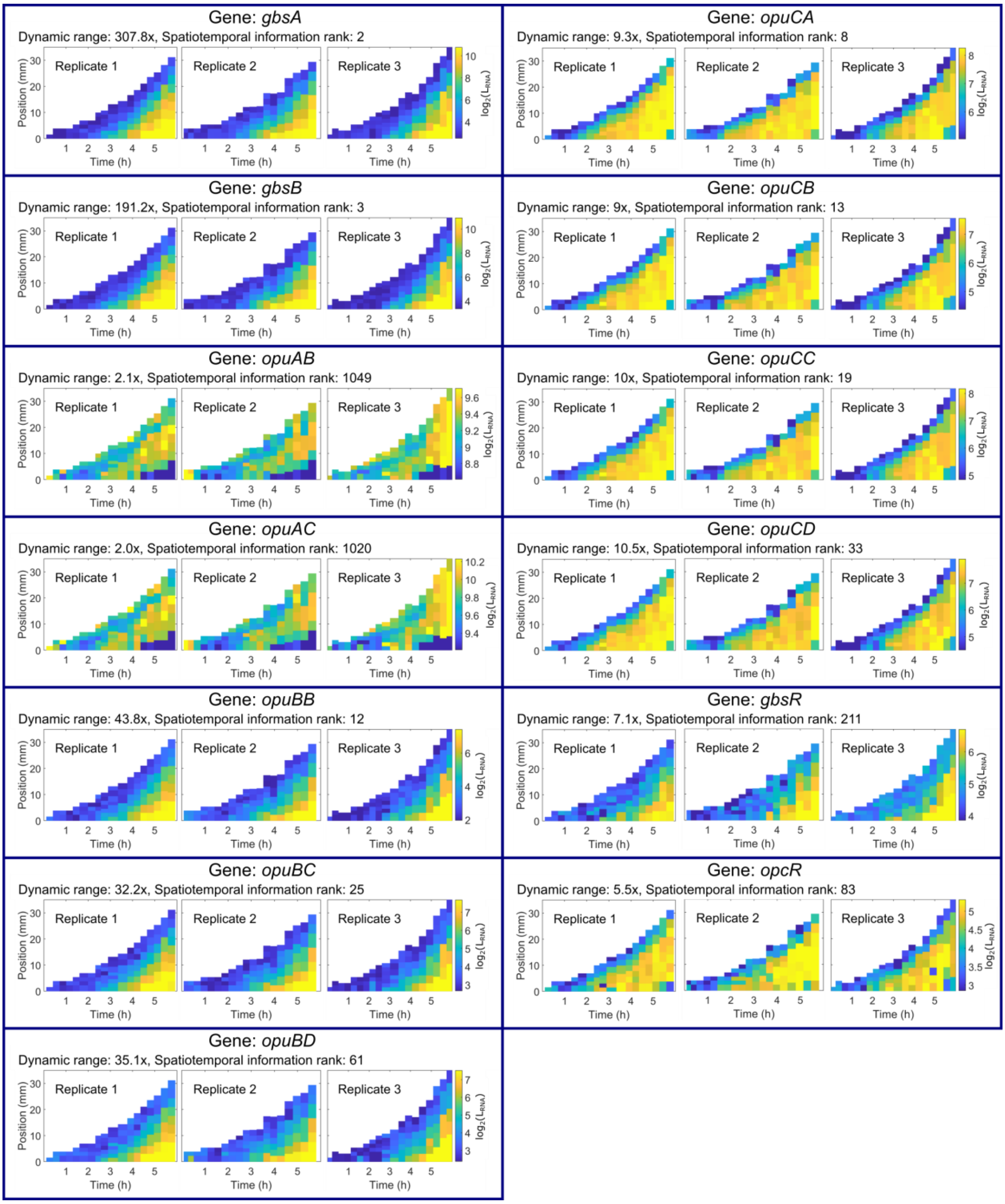

Figure S4: Osmolarity genes and their spatiotemporal expression. Spatiotemporal heatmaps show the gene expression during swarm development, and the colour of each tile in a heatmap indicates the expression level L_{RNA} of a particular gene. The dynamic range of a gene is defined as the ratio between the highest and the lowest colour bar value, which are the 95th and 5th percentile of gene expression values of all samples, respectively. The spatiotemporal information rank of a gene is introduced in Fig. 2b, where genes are ranked according to their spatiotemporal information score, with a higher rank value corresponding to lower spatiotemporal information. Shown are the spatiotemporal gene expression heatmaps of genes related to osmolarity. Differences in gene expression are clearly visible with high fold changes; many of the genes are upregulated in the late swarm centre.

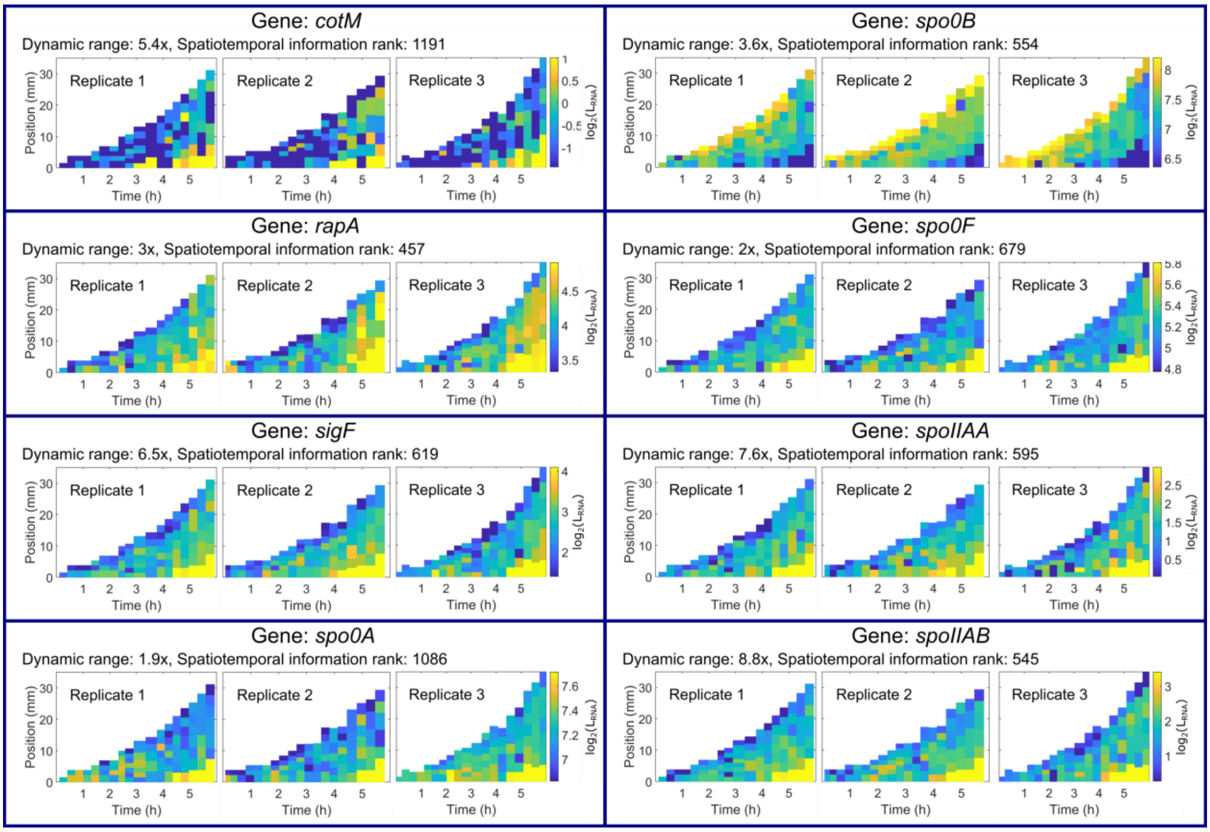

Figure S5: Sporulation genes and their spatiotemporal expression. Spatiotemporal heatmaps show the gene expression during swarm development, and the colour of each tile in a heatmap indicates the expression level L_{RNA} of a particular gene. The dynamic range of a gene is defined as the ratio between the highest and the lowest colour bar value, which are the 95th and 5th percentile of gene expression values of all samples, respectively. The spatiotemporal information rank of a gene is introduced in Fig. 2b, where genes are ranked according to their spatiotemporal information score, with a higher rank value corresponding to lower spatiotemporal information. Shown are the spatiotemporal gene expression heatmaps of genes related to sporulation. The master regulator for sporulation, *spo0A*, and forespore-specific sigma factor, *sigF*, are upregulated at the late swarm centre. Most genes are upregulated in the late swarm centre, with the exception of *spo0B*.

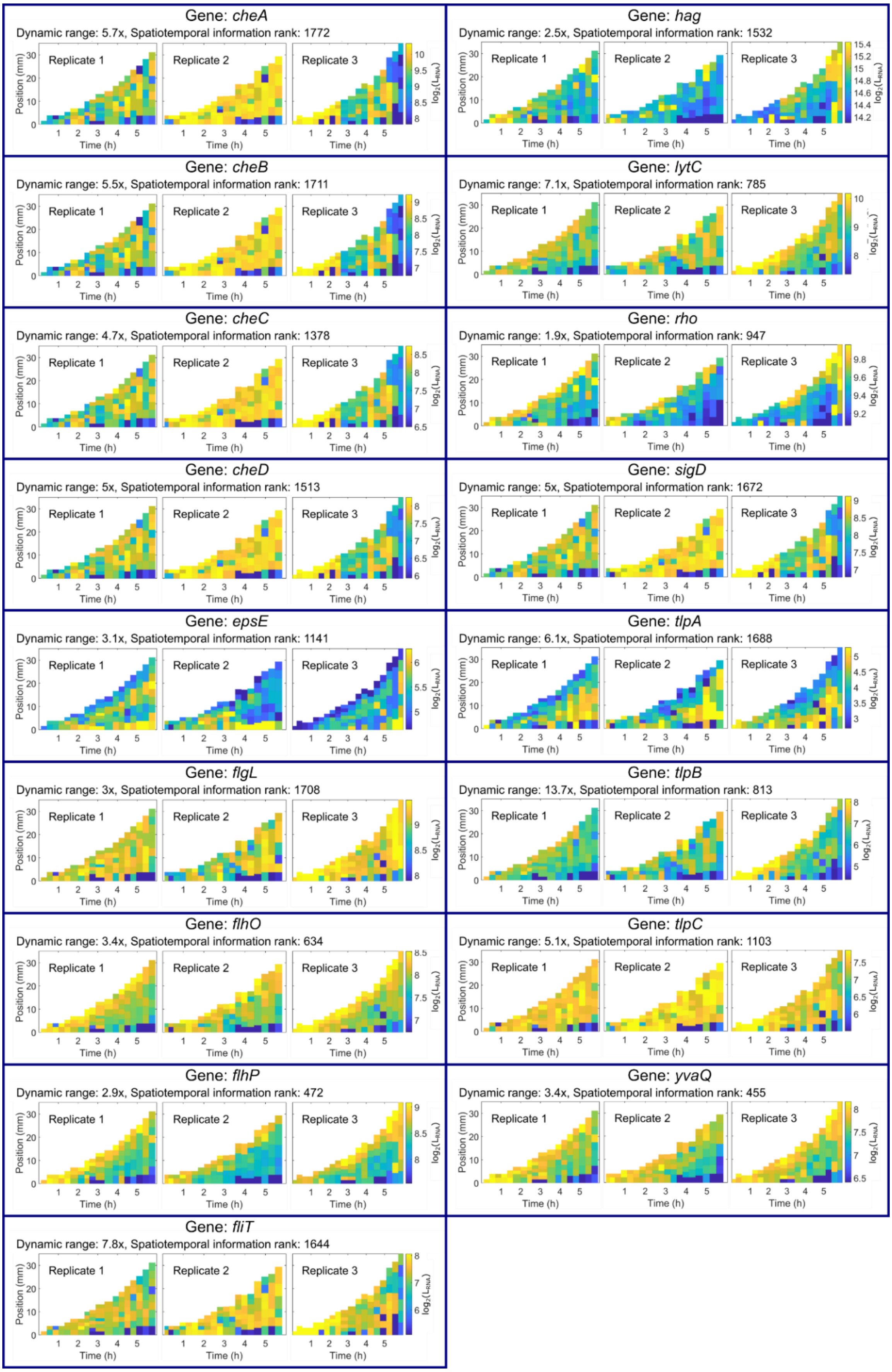

Figure S6: Motility genes and their spatiotemporal expression. Spatiotemporal heatmaps show the gene expression during swarm development, and the colour of each tile in a heatmap indicates the

expression level L_{RNA} of a particular gene. The dynamic range of a gene is defined as the ratio between the highest and the lowest colour bar value, which are the 95th and 5th percentile of gene expression values of all samples, respectively. The spatiotemporal information rank of a gene is introduced in Fig. 2b, where genes are ranked according to their spatiotemporal information score, with a higher rank value corresponding to lower spatiotemporal information. Shown are the spatiotemporal gene expression heatmaps of genes related to motility. Aside from a few exceptions, these genes are expressed higher at the swarm front and intermediate region, compared to the centre. This is consistent with observations of highly motile clusters close to the swarm front and non-motile cell chains at the swarm centre.

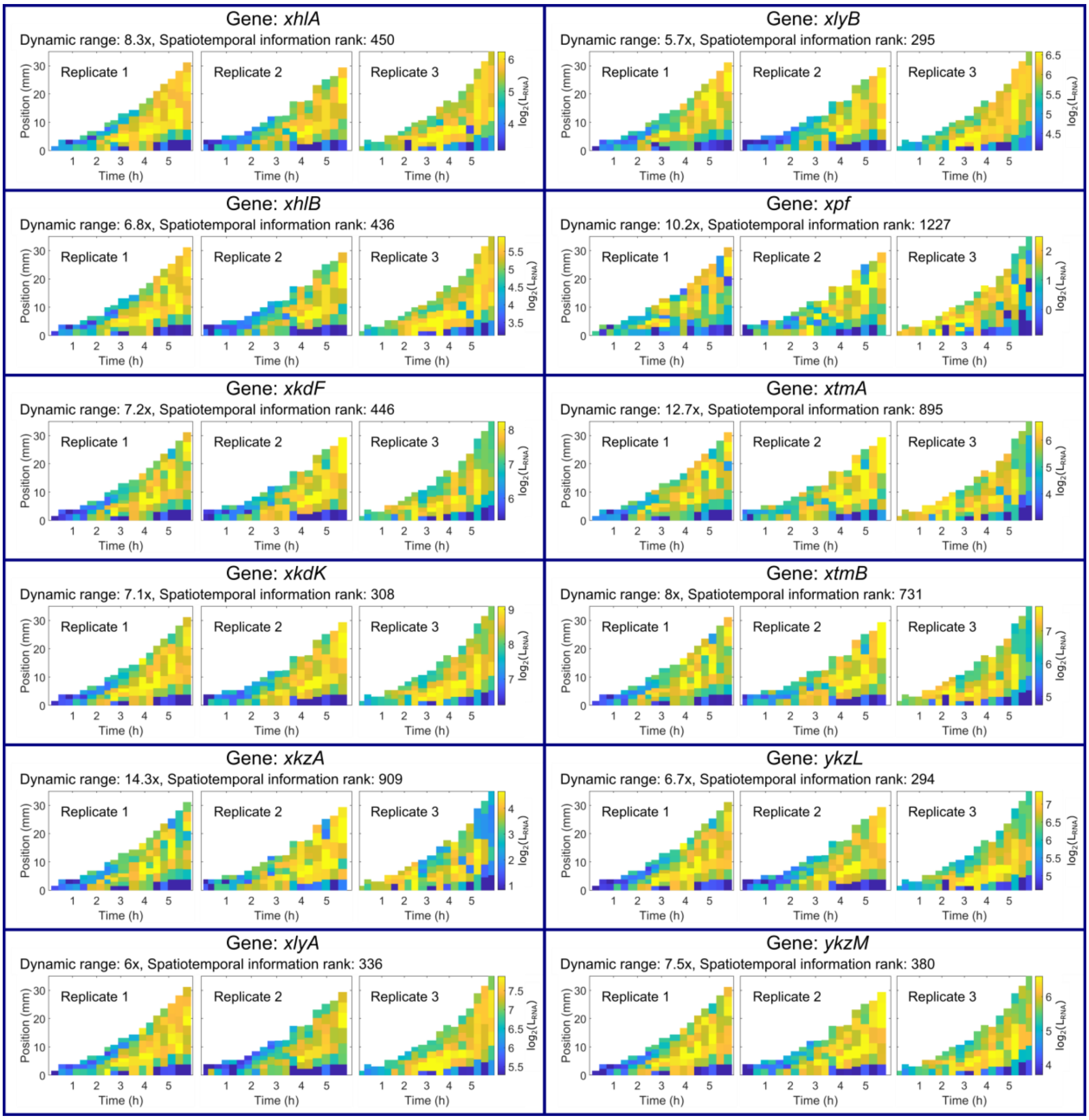

Figure S7: PBSX prophage genes and their spatiotemporal expression. Spatiotemporal heatmaps show the gene expression during swarm development, and the colour of each tile in a heatmap indicates the expression level L_{RNA} of a particular gene. The dynamic range of a gene is defined as the ratio between the highest and the lowest colour bar value, which are the 95th and 5th percentile of gene expression values of all samples, respectively. The spatiotemporal information rank of a gene is introduced in Fig. 2b, where genes are ranked according to their spatiotemporal information score, with a higher rank value corresponding to lower spatiotemporal information. Shown are the spatiotemporal gene expression heatmaps of genes related to the PBSX prophage, which all show a similar pattern: high expression in the intermediate region between the swarm front and the late swarm centre.

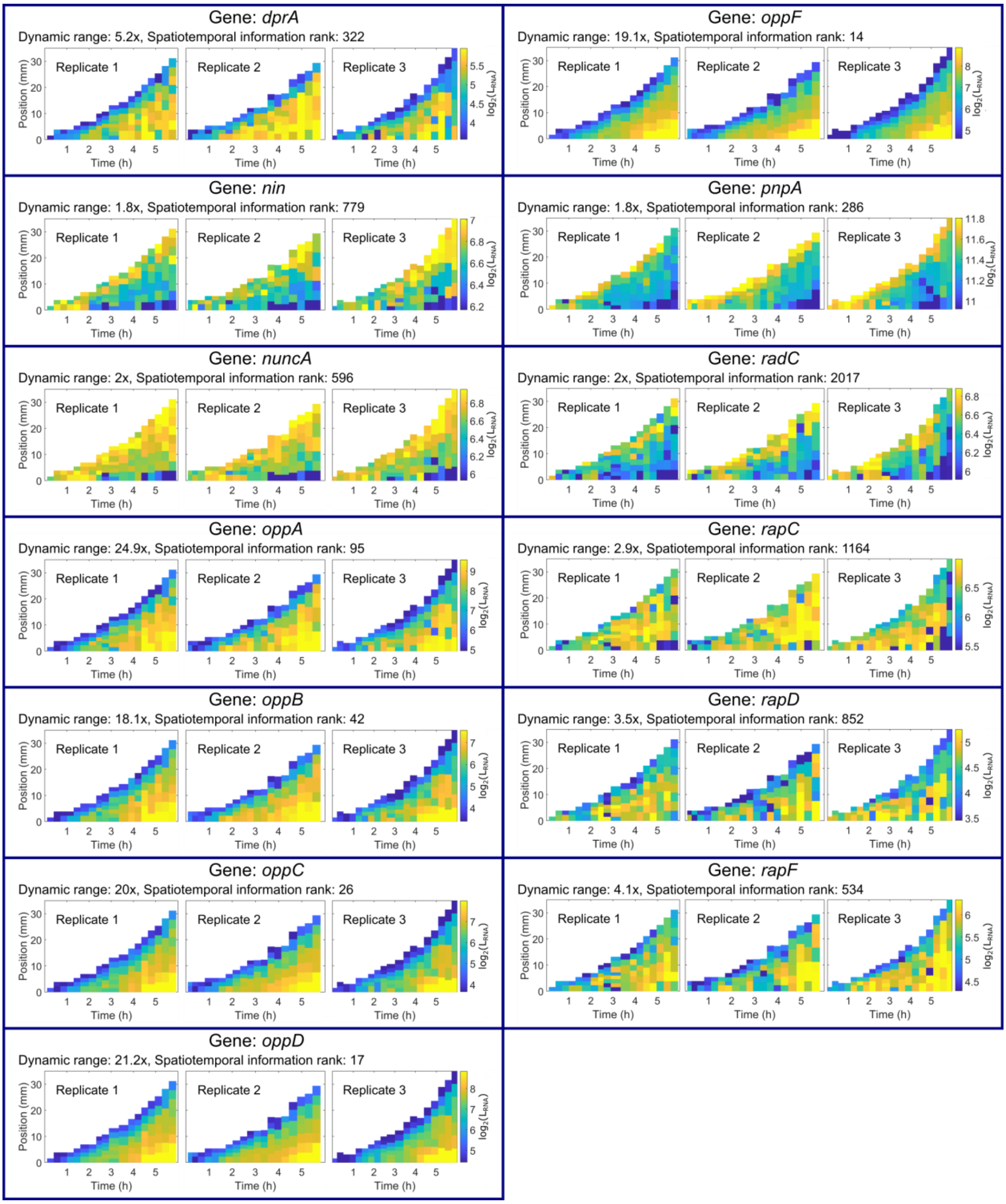

Figure S8: Competence genes and their spatiotemporal expression. Spatiotemporal heatmaps show the gene expression during swarm development, and the colour of each tile in a heatmap indicates the expression level L_{RNA} of a particular gene. The dynamic range of a gene is defined as the ratio between the highest and the lowest colour bar value, which are the 95th and 5th percentile of gene expression values of all samples, respectively. The spatiotemporal information rank of a gene is introduced in Fig. 2b, where genes are ranked according to their spatiotemporal information score, with a higher rank value corresponding to lower spatiotemporal information. Shown are the spatiotemporal gene expression heatmaps of genes related to competence. While specific patterns differ between genes in this figure, several genes show clear differential regulation in space and time with high fold-changed between swarm front and the late swarm centre.

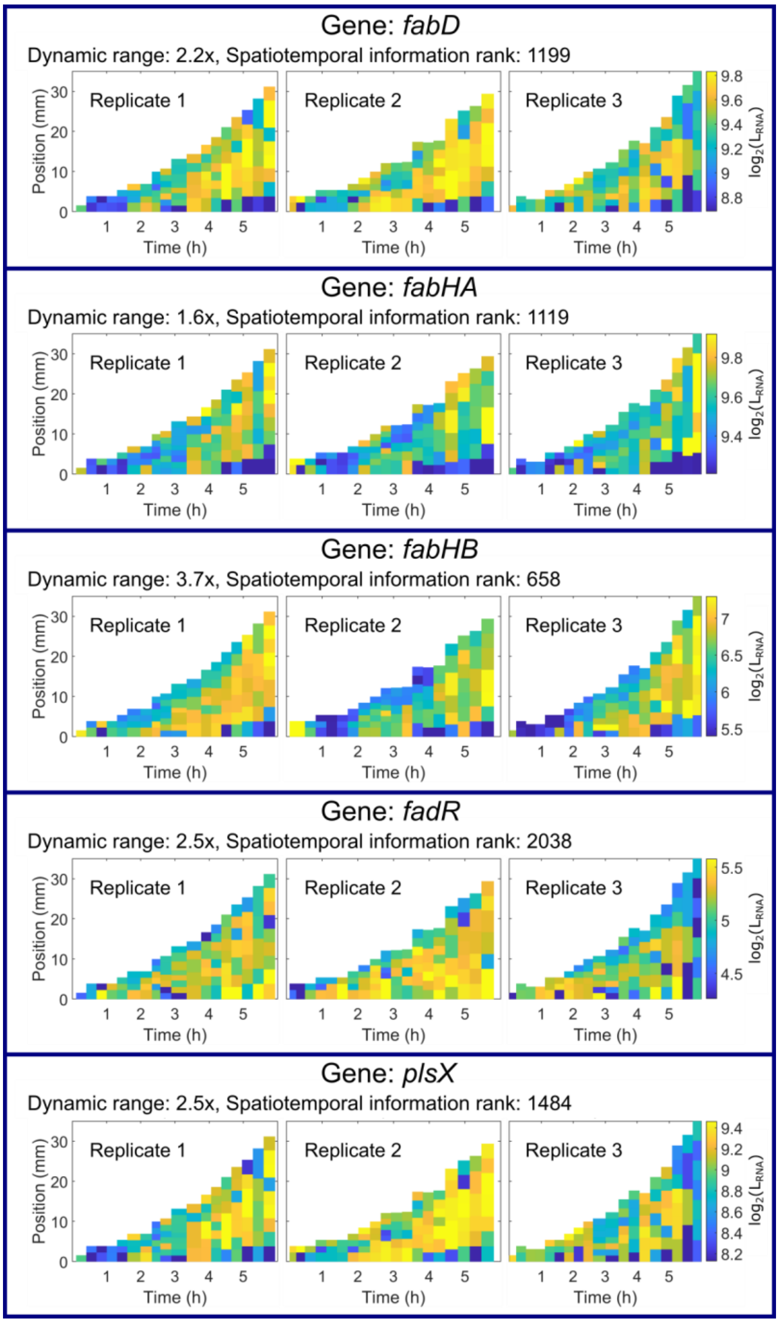

Figure S9: Fatty acid synthesis genes and their spatiotemporal expression. Spatiotemporal heatmaps show the gene expression during swarm development, and the colour of each tile in a heatmap indicates the expression level L_{RNA} of a particular gene. The dynamic range of a gene is defined as the ratio between the highest and the lowest colour bar value, which are the 95th and 5th percentile of gene expression values of all samples, respectively. The spatiotemporal information rank of a gene is introduced in Fig. 2b, where genes are ranked according to their spatiotemporal information score, with a higher rank value corresponding to lower spatiotemporal information. Shown are the spatiotemporal gene expression heatmaps of genes related to fatty acid synthesis. These genes show a pattern that is dissimilar from most expression patterns, where the highest expression is observable in the intermediate region between the swarm front and the swarm centre.

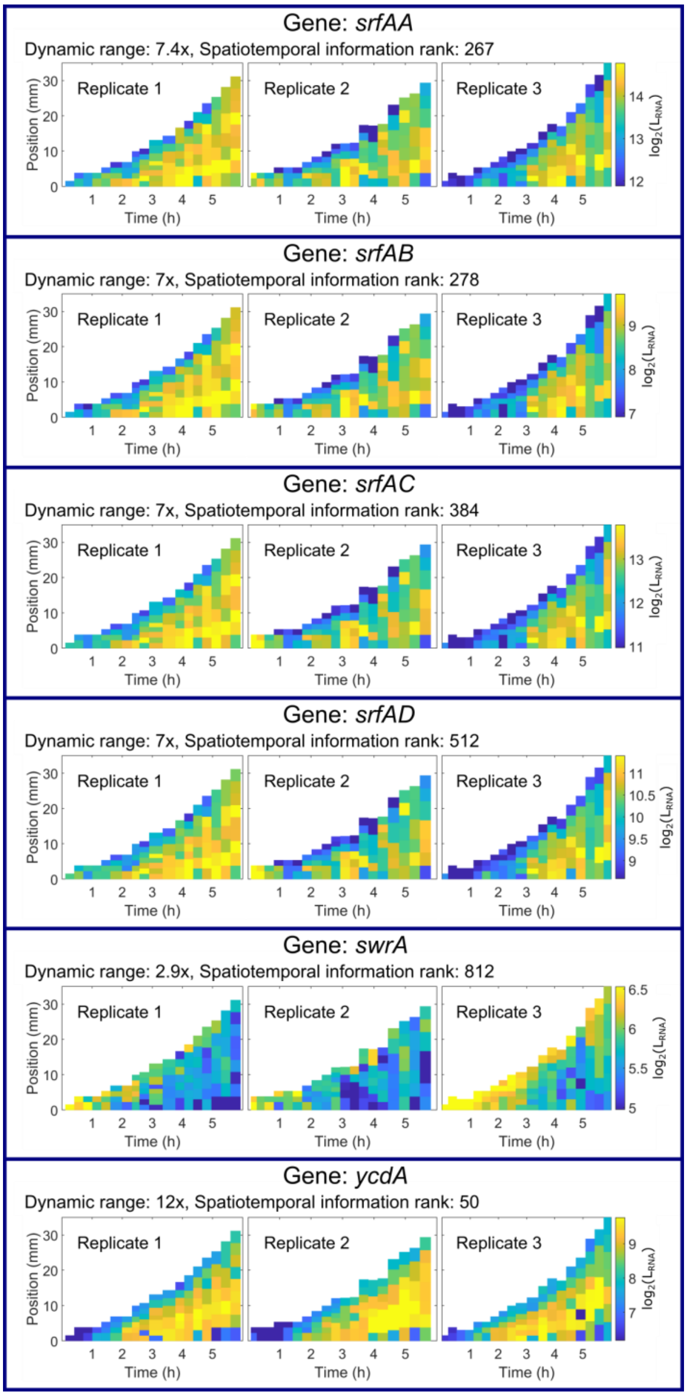

Figure S10: Swarming genes and their spatiotemporal expression. Spatiotemporal heatmaps show the gene expression during swarm development, and the colour of each tile in a heatmap indicates the expression level L_{RNA} of a particular gene. The dynamic range of a gene is defined as the ratio between the highest and the lowest colour bar value, which are the 95th and 5th percentile of gene expression values of all samples, respectively. The spatiotemporal information rank of a gene is introduced in Fig. 2b, where genes are ranked according to their spatiotemporal information score, with a higher rank value corresponding to lower spatiotemporal information. Shown are the spatiotemporal gene expression heatmaps of genes known to be essential for *B. subtilis* swarming. Most genes show highest expression at the intermediate region, where fatty acid synthesis is also upregulated (see Fig. S9), suggesting that fatty acids synthesized in the intermediate region between the swarm front and swarm centre might be used as a substrate for surfactin production by *srfAA*–*srfAD* genes.

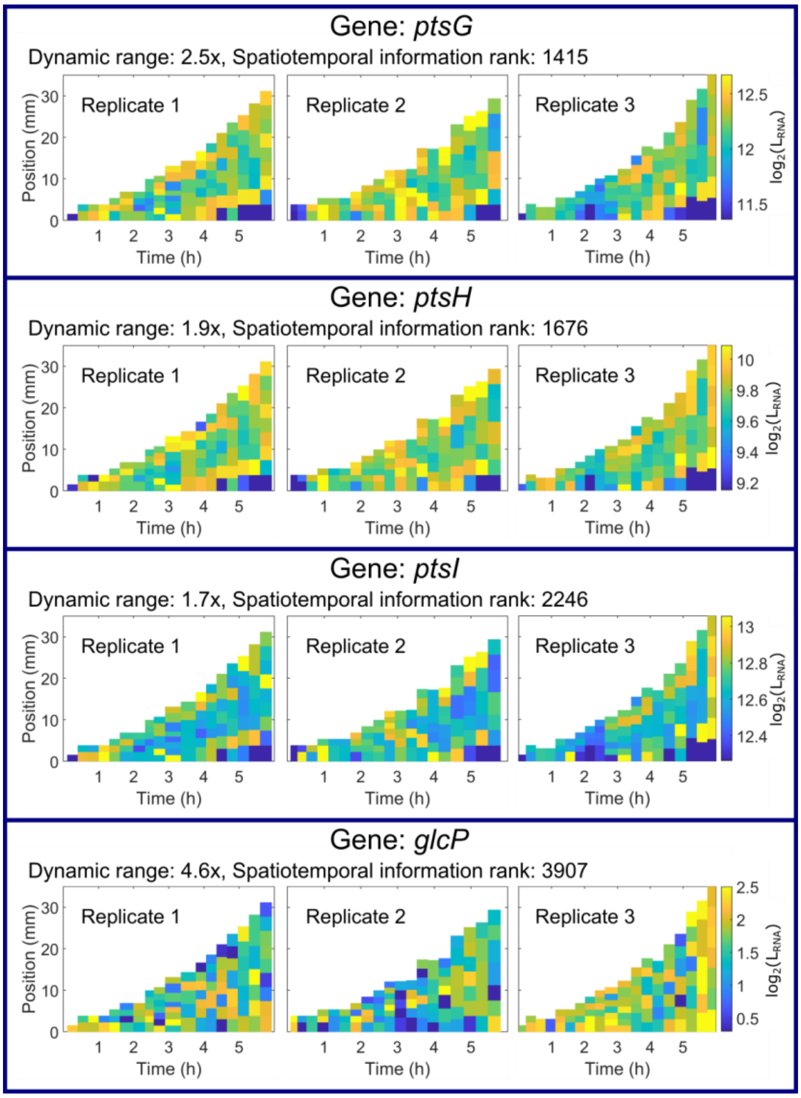

Figure S11: Glucose import genes and their spatiotemporal expression. Spatiotemporal heatmaps show the gene expression during swarm development, and the colour of each tile in a heatmap indicates the expression level L_{RNA} of a particular gene. The dynamic range of a gene is defined as the ratio between the highest and the lowest colour bar value, which are the 95th and 5th percentile of gene expression values of all samples, respectively. The spatiotemporal information rank of a gene is introduced in Fig. 2b, where genes are ranked according to their spatiotemporal information score, with a higher rank value corresponding to lower spatiotemporal information. Shown are the normalized expression levels of the glucose-specific PTS genes *ptsGHI* and non-PTS type glucose transporters *glcP.* Expression levels of the non-PTS glucose transporter *glcU* were too low to pass our quality criterion (more than 10 reads in at least 2 samples), which is why this gene was not further analysed and is not shown here. The four genes displayed here do not show any strong spatiotemporal pattern, indicating that they are not strongly regulated during *B. subtilis* swarm expansion on soft LB agar.

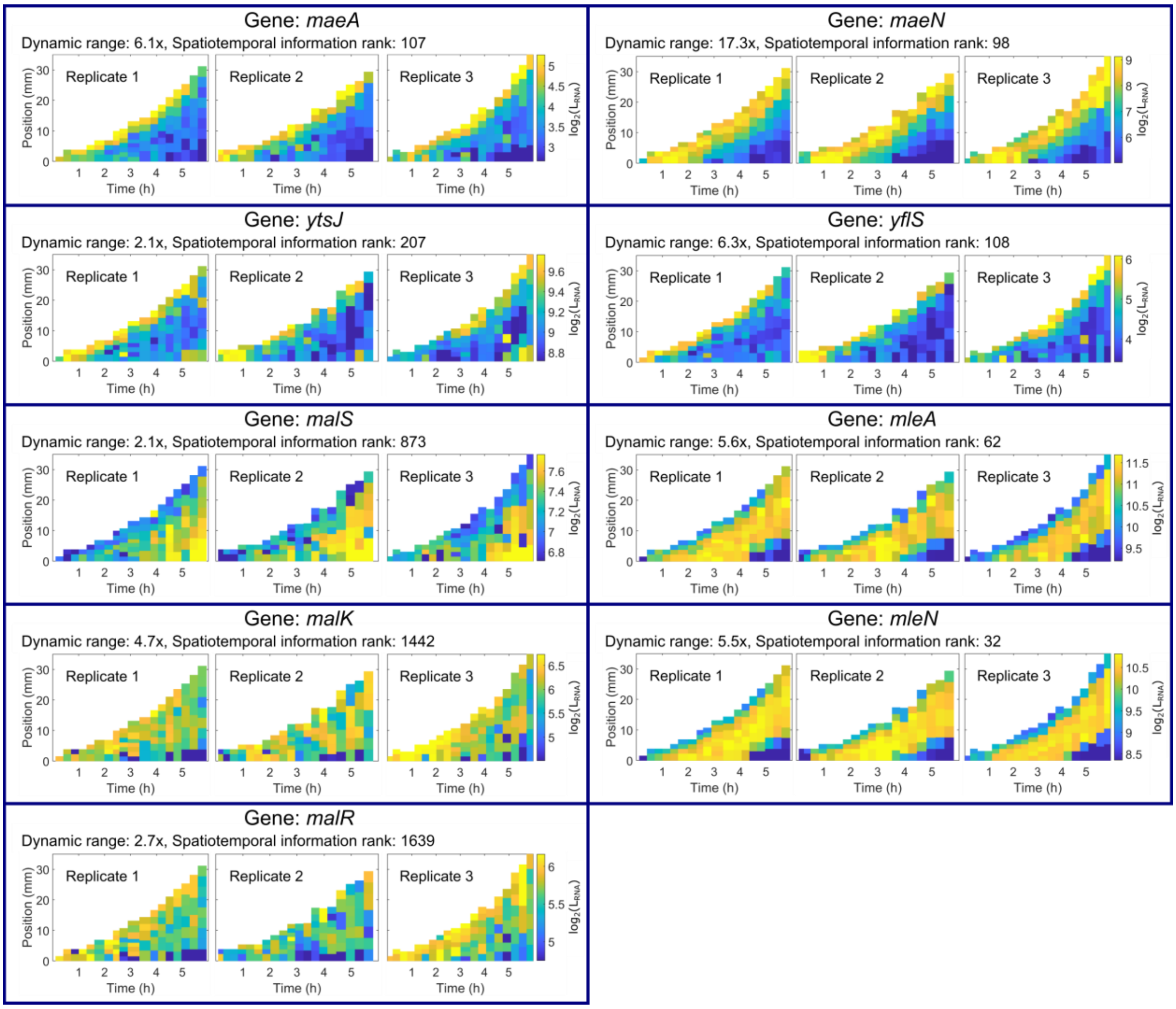

Figure S12: Malate metabolism additional genes and their spatiotemporal expression. Spatiotemporal heatmaps show the gene expression during swarm development, and the colour of each tile in a heatmap indicates the expression level L_{RNA} of a particular gene. The dynamic range of a gene is defined as the ratio between the highest and the lowest colour bar value, which are the 95th and 5th percentile of gene expression values of all samples, respectively. The spatiotemporal information rank of a gene is introduced in Fig. 2b, where genes are ranked according to their spatiotemporal information score, with a higher rank value corresponding to lower spatiotemporal information. Shown are the expression levels of the *maeLM* two-component system, malate transporter *maeS*, and the malate symporter *maeN*. The *maeLM* two-component system activates transcription of *maeS, maeN*, and the malic enzyme *maeA* in response to the presence of malate. Schematic spatiotemporal gene expression heatmaps of some of these and further genes related to malate and carbon metabolism, are shown in the main text Fig. 3.

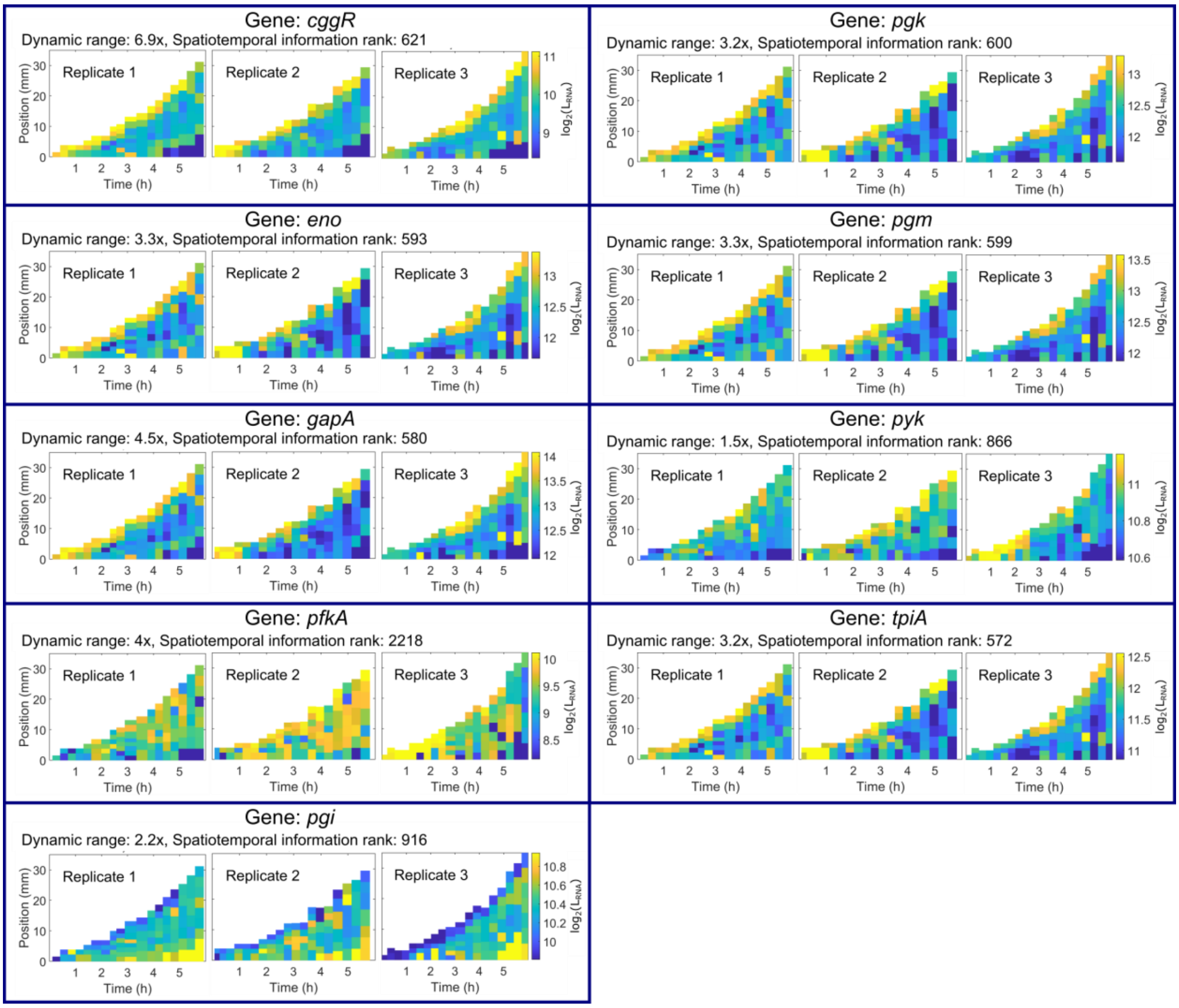

Figure S13: Glycolysis genes and their spatiotemporal expression. Spatiotemporal heatmaps show the gene expression during swarm development, and the colour of each tile in a heatmap indicates the expression level L_{RNA} of a particular gene. The dynamic range of a gene is defined as the ratio between the highest and the lowest colour bar value, which are the 95th and 5th percentile of gene expression values of all samples, respectively. The spatiotemporal information rank of a gene is introduced in Fig. 2b, where genes are ranked according to their spatiotemporal information score, with a higher rank value corresponding to lower spatiotemporal information. Shown are the expression levels of genes involved in glycolysis with a distinct spatiotemporal patterns. The majority of genes are upregulated at the swarm front compared to the swarm centre. The gene *pgi,* encoding glucose-6-phosphate isomerase, has an exceptional pattern, which might reflect elevated production of glucose 6-phosphate derived from maltose at the swarm centre (see Fig. S19).

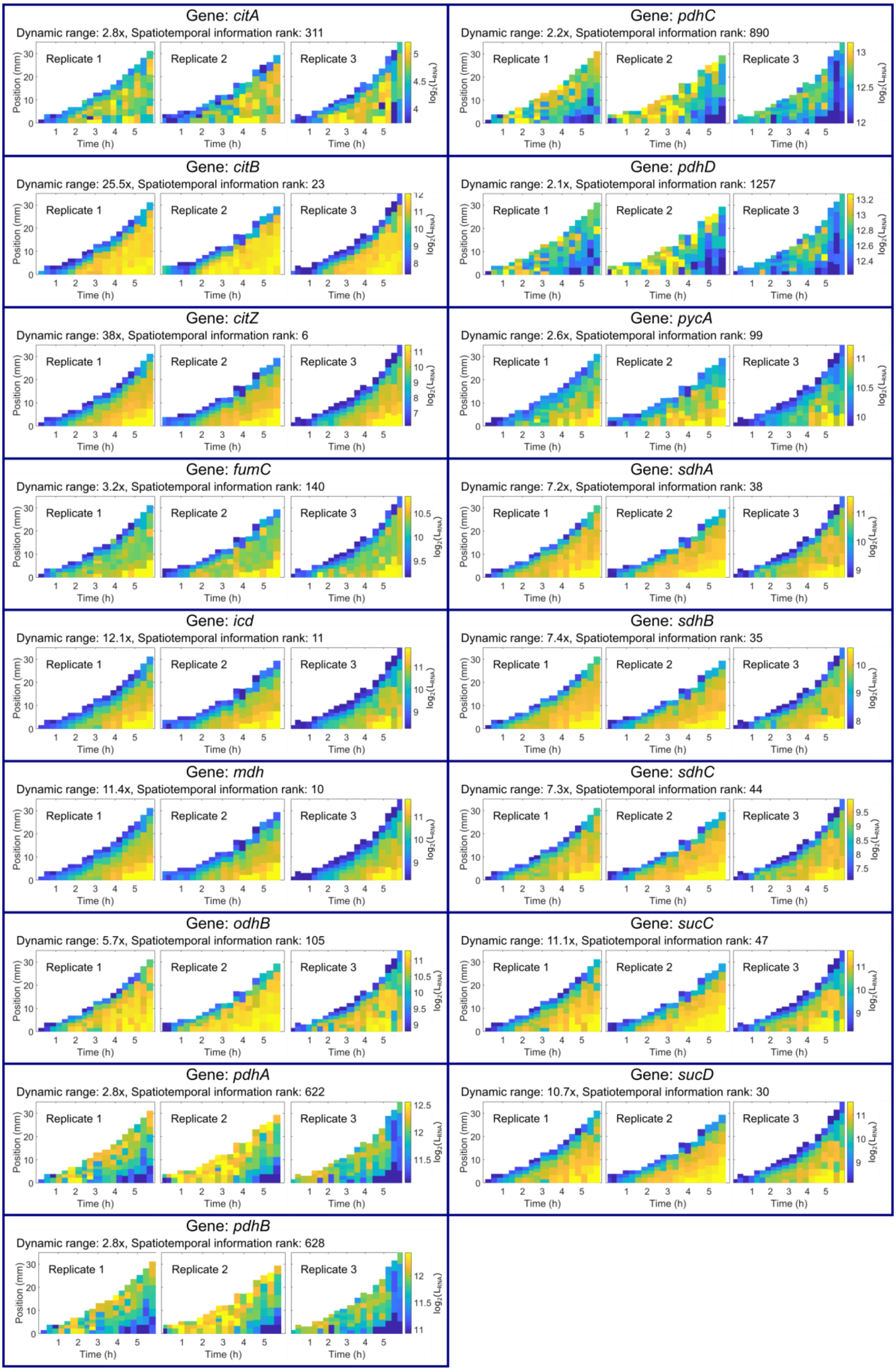

Figure S14: TCA cycle genes and their spatiotemporal expression. Spatiotemporal heatmaps show the gene expression during swarm development, and the colour of each tile in a heatmap indicates

the expression level L_{RNA} of a particular gene. The dynamic range of a gene is defined as the ratio between the highest and the lowest colour bar value, which are the 95th and 5th percentile of gene expression values of all samples, respectively. The spatiotemporal information rank of a gene is introduced in Fig. 2b, where genes are ranked according to their spatiotemporal information score, with a higher rank value corresponding to lower spatiotemporal information. All genes directly involved in the TCA cycle are strongly upregulated towards the late swarm center, indicating that the TCA cycle is most active in this region. *pdhABCD* genes that convert pyruvate to acetyl-CoA are upregulated at the swarm front and the intermediate regions rather than the swarm centre, supporting the hypothesis that *B. subtilis* cells perform overflow metabolism at the swarm front. The *pycA* gene, which is involved in the replenishment of the oxaloacetate pool by converting pyruvate to oxaloacetate, is upregulated in the late swarm centre, implying that pyruvate produced by overflow metabolism is re-used by the cells and metabolized to fuel the TCA cycle.

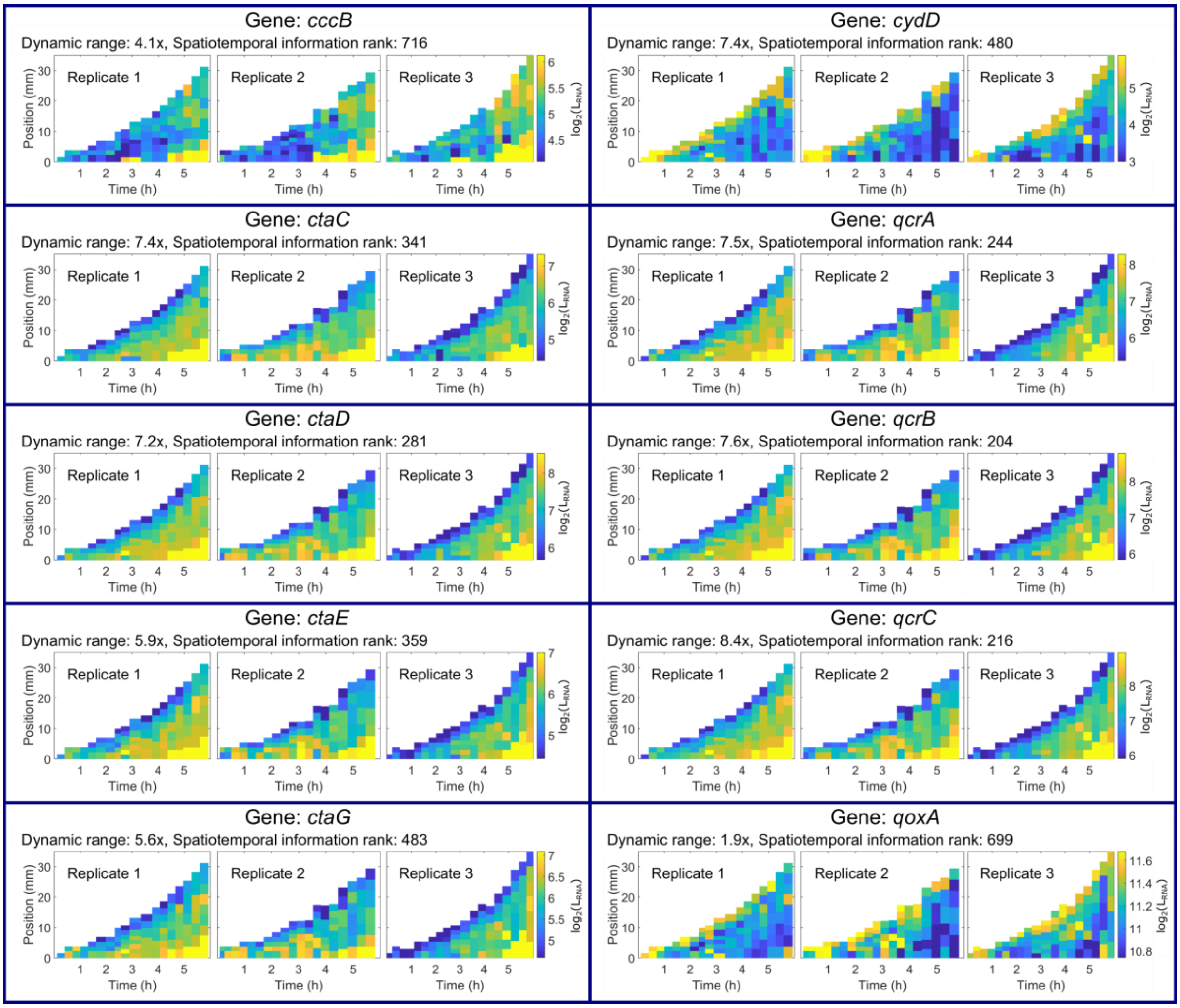

Figure S15: Respiration genes and their spatiotemporal expression. Spatiotemporal heatmaps show the gene expression during swarm development, and the colour of each tile in a heatmap indicates the expression level L_{RNA} of a particular gene. The dynamic range of a gene is defined as the ratio between the highest and the lowest colour bar value, which are the 95th and 5th percentile of gene expression values of all samples, respectively. The spatiotemporal information rank of a gene is introduced in Fig. 2b, where genes are ranked according to their spatiotemporal information score, with a higher rank value corresponding to lower spatiotemporal information. Shown are the spatiotemporal gene expression heatmaps of genes related to respiration. Most genes have a higher expression in the late swarm centre compared to the swarm front, implying the shift of energy acquisition modes from substrate-level phosphorylation at the swarm front to oxidative phosphorylation at the swarm centre.

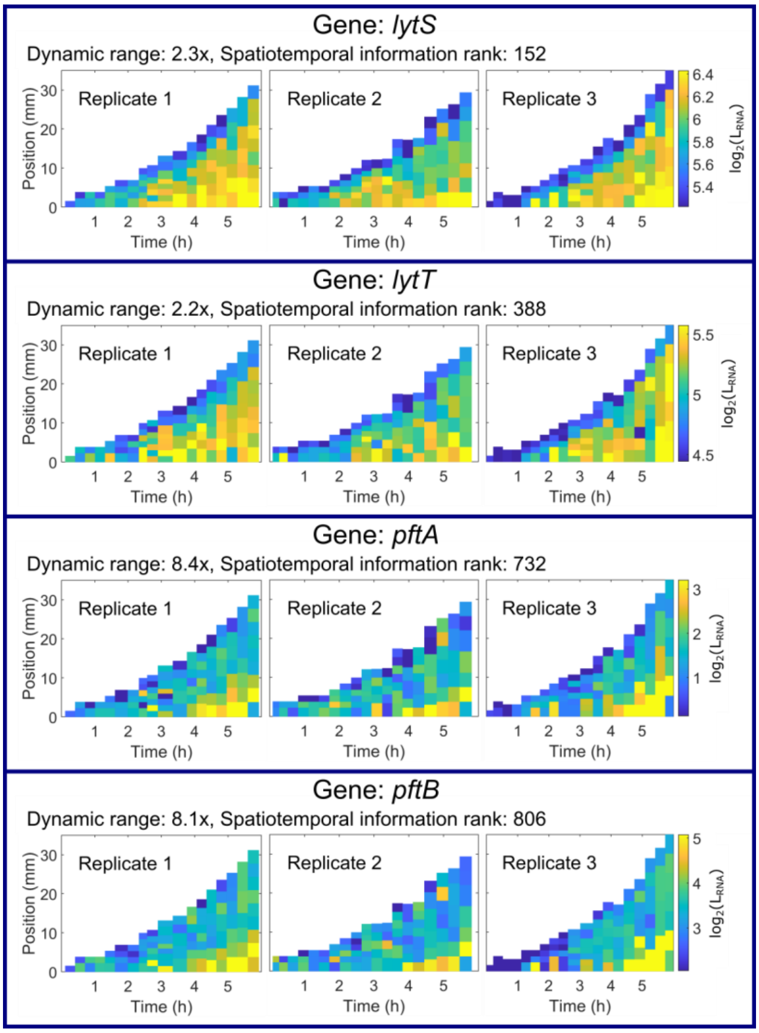

Figure S16: Two-component system for pyruvate utilization and its spatiotemporal expression. Spatiotemporal heatmaps show the gene expression during swarm development, and the colour of each tile in a heatmap indicates the expression level L_{RNA} of a particular gene. The dynamic range of a gene is defined as the ratio between the highest and the lowest colour bar value, which are the 95th and 5th percentile of gene expression values of all samples, respectively. The spatiotemporal information rank of a gene is introduced in Fig. 2b, where genes are ranked according to their spatiotemporal information score, with a higher rank value corresponding to lower spatiotemporal information. Shown are the expression levels of *lytS* and *lytT*, a two-component sensor kinase and response regulator, controlling pyruvate utilization. Both are upregulated in the late swarm centre compared to the swarm front, which implies that pyruvate is predominantly utilized in the swarm centre.

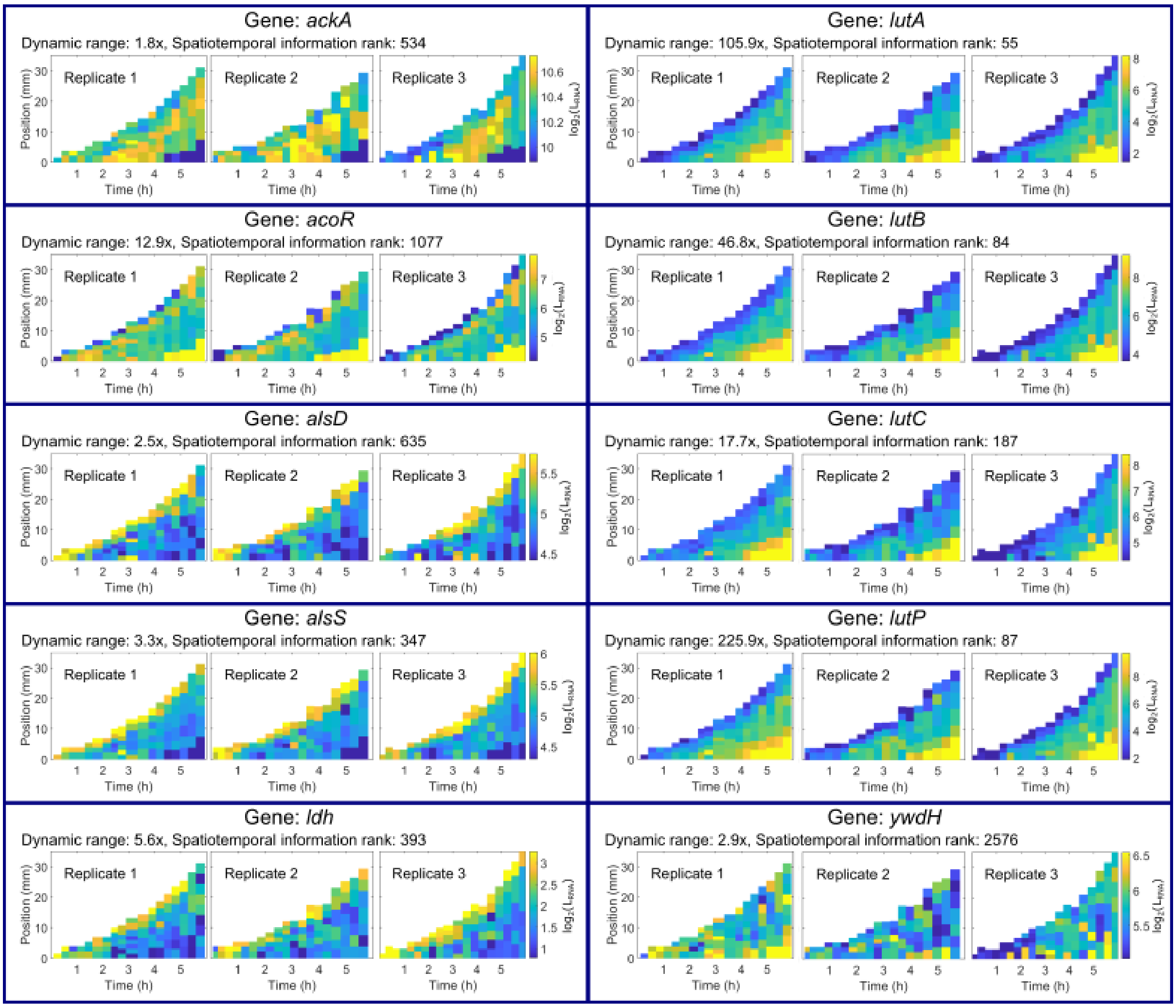

Figure S17: Fermentation genes and their spatiotemporal expression. Spatiotemporal heatmaps show the gene expression during swarm development, and the colour of each tile in a heatmap indicates the expression level L_{RNA} of a particular gene. The dynamic range of a gene is defined as the ratio between the highest and the lowest colour bar value, which are the 95th and 5th percentile of gene expression values of all samples, respectively. The spatiotemporal information rank of a gene is introduced in Fig. 2b, where genes are ranked according to their spatiotemporal information score, with a higher rank value corresponding to lower spatiotemporal information. Shown are the spatiotemporal gene expression heatmaps of genes involved in acetate, acetoin, and lactate fermentation. *B. subtilis* cells perform overflow metabolism at the swarm front as *alsDS* are upregulated, leading to the accumulation of pyruvate and fermentation products such as acetate, acetoin, and lactate. Additional experiments (main text Fig. 4) indicate that some of those products are re-metabolized by the next generation of cells that inhabit the intermediate region and the swarm centre. This is consistent with the pyruvate two-component system (Fig. S16), lactate permease gene *lutP*, and the fact that lactate utilization genes *lutABC* are upregulated in these regions.

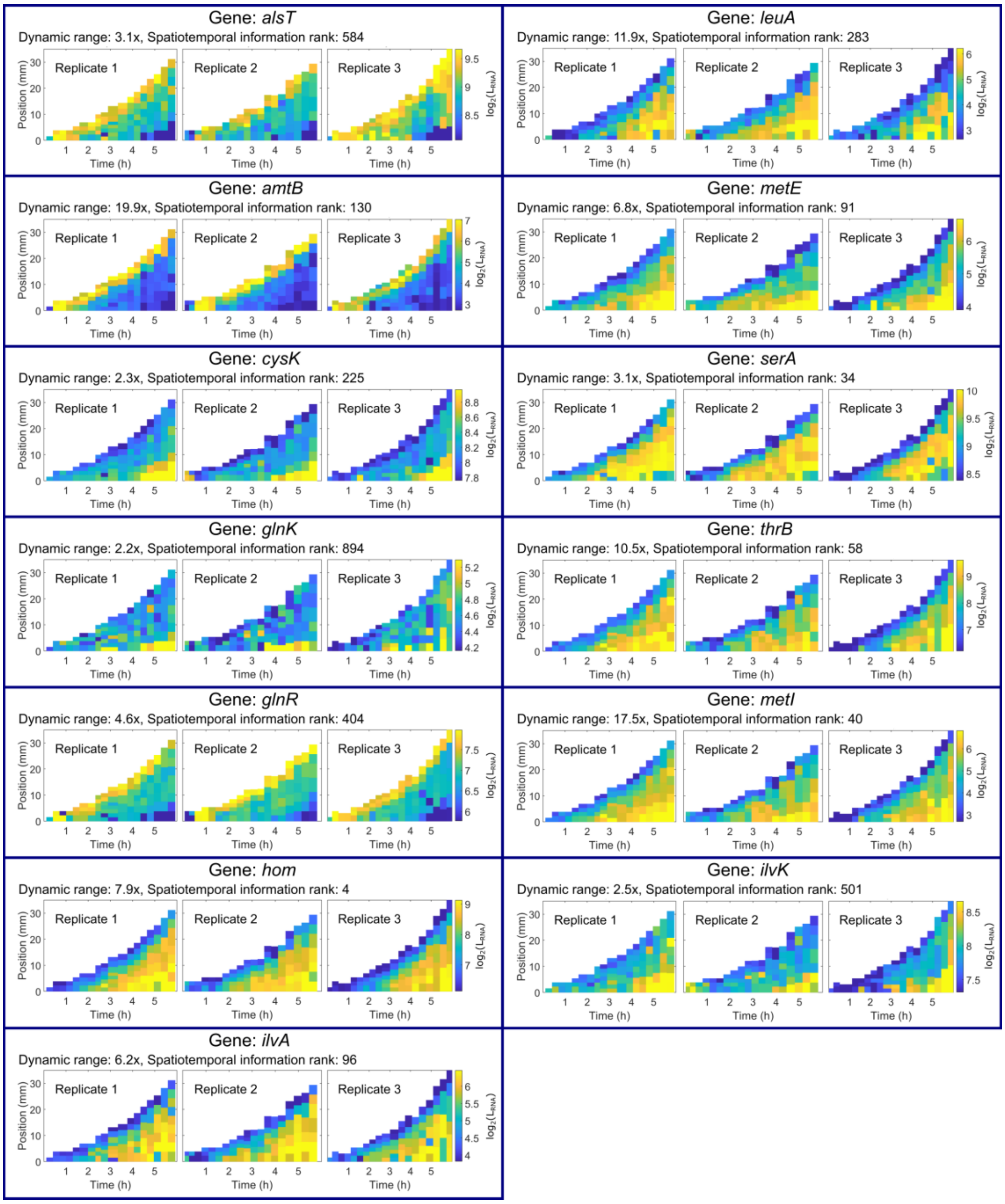

Figure S18: Amino acid uptake and synthesis genes and their spatiotemporal expression. Spatiotemporal heatmaps show the gene expression during swarm development, and the colour of each tile in a heatmap indicates the expression level L_{RNA} of a particular gene. The dynamic range of a gene is defined as the ratio between the highest and the lowest colour bar value, which are the 95th and 5th percentile of gene expression values of all samples, respectively. The spatiotemporal information rank of a gene is introduced in Fig. 2b, where genes are ranked according to their spatiotemporal information score, with a higher rank value corresponding to lower spatiotemporal information. Shown are the spatiotemporal gene expression heatmaps of genes related to the uptake and synthesis of amino acids. Genes related to amino acid uptake, *alsT*, *amtB, glnK,* and *glnR,* are upregulated at the swarm front while genes involved in the synthesis of amino acids, *hom, ilvA, ilvK, leuA, metE, serA, thrB*, and *metI*, are strongly upregulated at the late swarm centre, which implies cells at the swarm front consume amino acids coming from tryptone within the LB medium, whereas the next generation of cells (in the intermediate region and the swarm centre) have to synthesize amino acids, especially at the swarm centre.

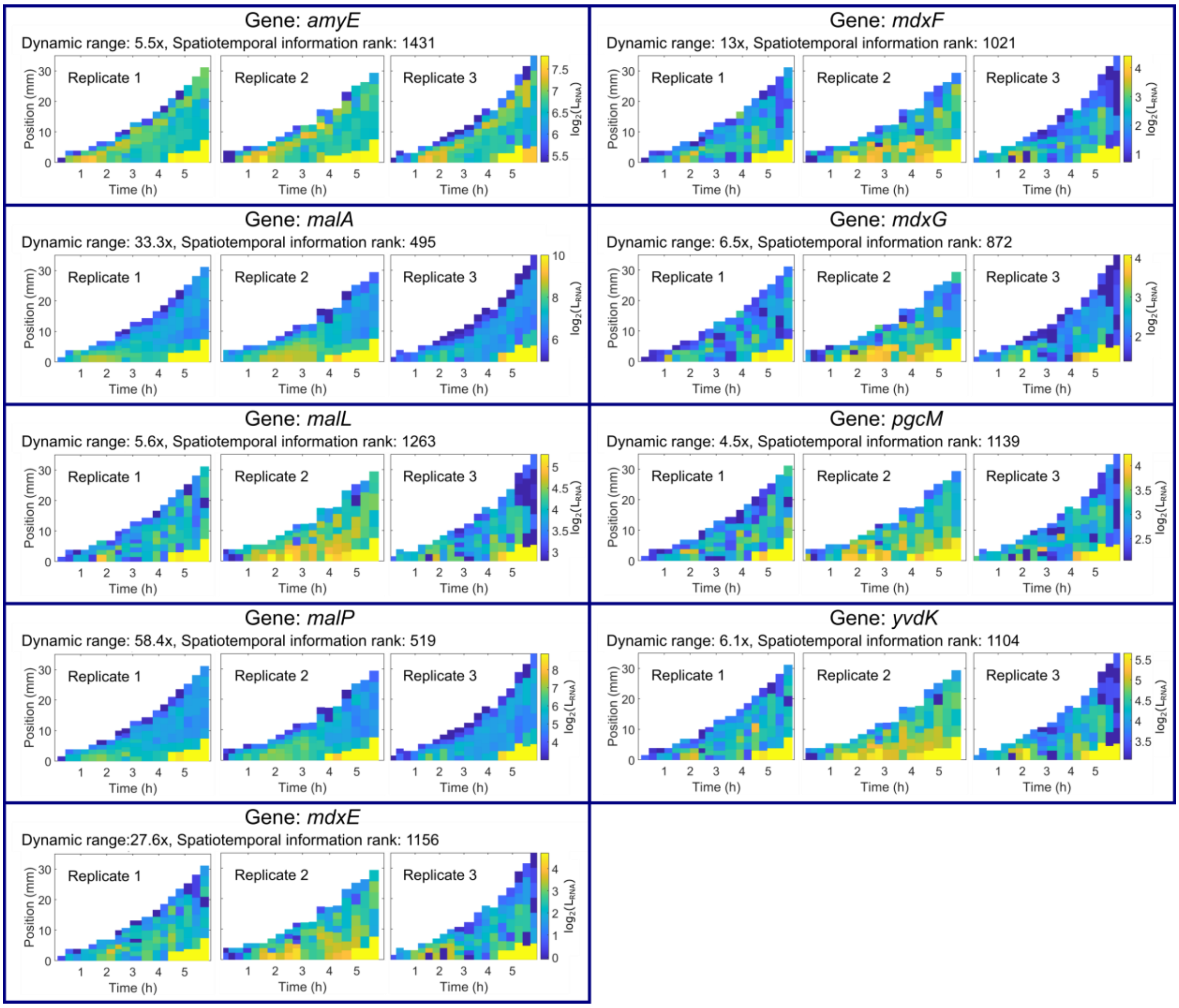

Figure S19: Maltose and maltodextrin utilization genes and their spatiotemporal expression. Spatiotemporal heatmaps show the gene expression during swarm development, and the colour of each tile in a heatmap indicates the expression level L_{RNA} of a particular gene. The dynamic range of a gene is defined as the ratio between the highest and the lowest colour bar value, which are the 95th and 5th percentile of gene expression values of all samples, respectively. The spatiotemporal information rank of a gene is introduced in Fig. 2b, where genes are ranked according to their spatiotemporal information score, with a higher rank value corresponding to lower spatiotemporal information. Shown are the expression levels of genes related to the uptake and degradation of maltose and maltodextrin. The *amyE* gene encodes alpha-amylase, which degrades polymeric carbohydrates such as glycogen to maltodextrin and maltose. *mdxEFG* genes encode maltodextrin ABC transporter. The *yvdK*, *malL,* and *pgcM* genes are involved in maltodextrin degradation. The *malA-glvR-malP* operon is involved in the uptake and metabolism of maltose. All of these genes are upregulated at the late swarm centre, implying that there is a supply of polymeric carbohydrates in this region.

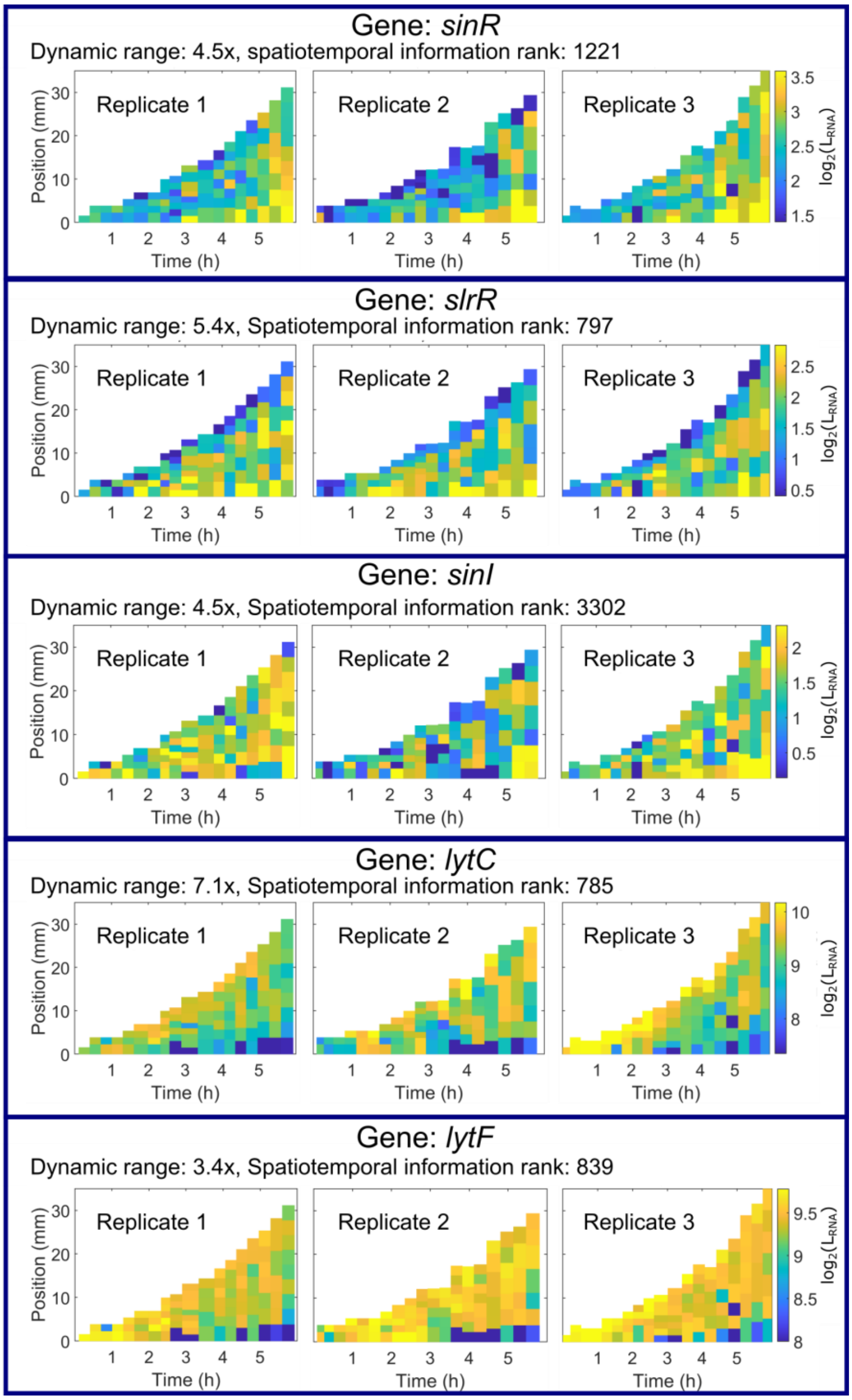

Figure S20: Expression patterns of genes related to cell chaining. Spatiotemporal heatmaps show the gene expression during swarm development, and the colour of each tile in a heatmap indicates the expression level L_{RNA} of a particular gene. The dynamic range of a gene is defined as the ratio between the highest and the lowest colour bar value, which are the 95th and 5th percentile of gene expression values of all samples, respectively. The spatiotemporal information rank of a gene is introduced in Fig. 2b, where genes are ranked according to their spatiotemporal information score, with a higher rank value corresponding to lower spatiotemporal information. Shown are the expression levels of *sinR*, *slrR* and *sinI*, which are genes associated with the SinR-SlrR complex that can switch the cellular state between a normal cell morphology and the formation of cell chains[1]. The SinR-SlrR complex represses *lytC* and *lytF* that encode autolysins, leading to the formation of cell chaining. While *sinR*, *slrR* and *sinI* are relatively noisy and therefore difficult to interpret, *lytC* and *lytF* show clear patterns of downregulation in the swarm centre, consistent with cell chaining previously reported[2].

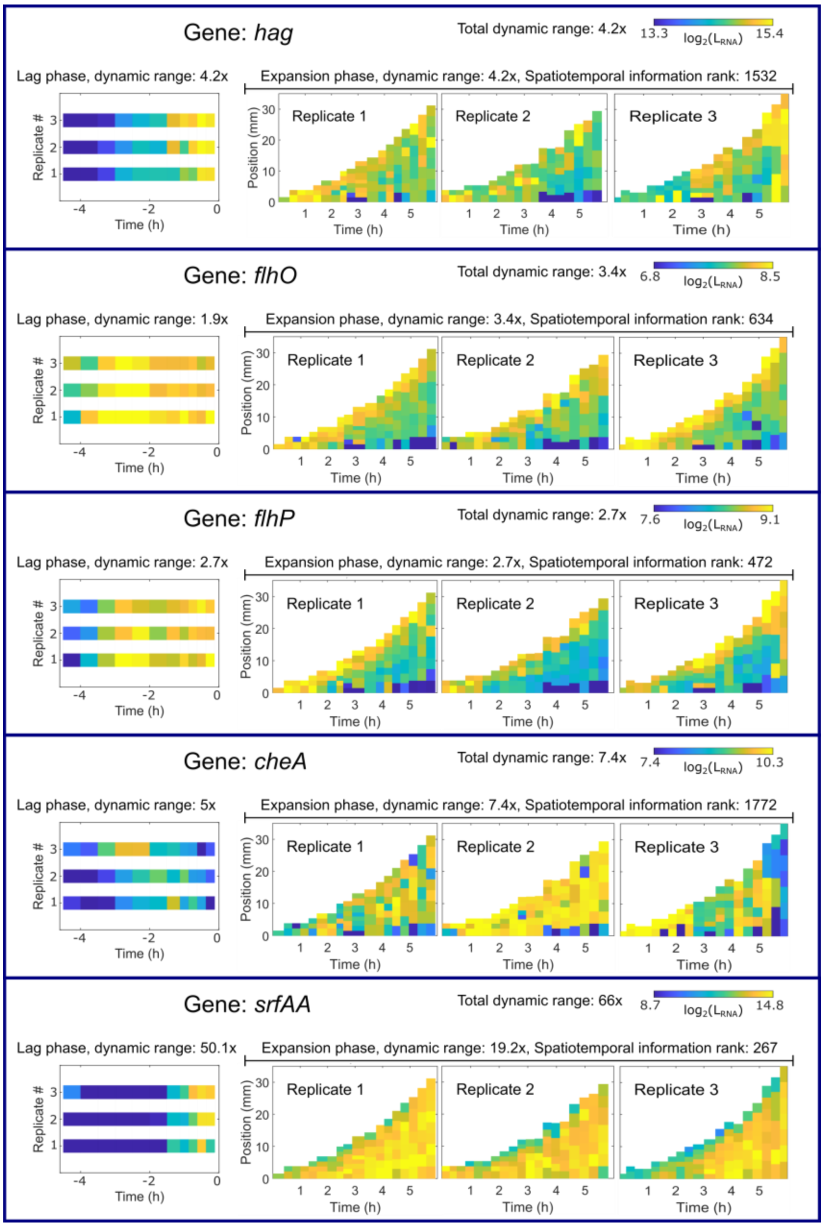

Figure S21: Expression patterns during the lag phase and the swarm expansion phase, for selected motility genes. Heatmaps on the left show lag phase gene expression for three replicates over a course of 4.5 hours preceding the swarm expansion phase. Heatmaps on the right display spatiotemporal gene expression during the swarm expansion phase. All heatmaps within a row share the same colour bar. Total dynamic range of a gene is defined as the ratio between the lowest and highest value of the colour bar, which are the 5th and 95th percentile of combined lag phase and expansion phase samples. The dynamic ranges displayed above the lag phase and expansion phase heatmaps only take samples into account which belong to each phase respectively. The spatiotemporal information rank of a gene is introduced in Fig. 2b, where genes are ranked according to their spatiotemporal information score, with a higher rank value corresponding to lower spatiotemporal information. Note that in this figure, the normalization of the transcriptome data differs from the normalization that was used for all other figures in this article – therefore the numerical values shown in this figure are not the same as those shown in Fig. 1e, Fig. S6, and Fig. S10. The difference in normalization is that in this figure, the data were normalized using the samples of the expansion phase and the lag phase, whereas in all other figures in this article, the data were only normalized among the expansion phase samples.

References in the Supplementary Information

1. Chai, Y., Kolter, R. & Losick, R. Reversal of an epigenetic switch governing cell chaining in Bacillus subtilis by protein instability. *Mol. Microbiol.* **78**, 218–229 (2010).
2. Kearns, D. B. & Losick, R. Swarming motility in undomesticated Bacillus subtilis. *Mol. Microbiol.* **49**, 581–590 (2003).
