# Supplementary material for: Simultaneous spatiotemporal transcriptomics and microscopy of Bacillus subtilis swarm development reveal cooperation across generations
Source: Nat Microbiol. 2023 Nov 16;8(12):2378–91. doi: 10.1038/s41564-023-01518-4 (PMC10686836; doi:10.1038/s41564-023-01518-4)
Supplement: Supplementary file 2 — Reporting Summary [file 41564_2023_1518_MOESM2_ESM.pdf]

nature portfolio

Corresponding author(s): Knut Drescher
Last updated by author(s): Oct 3, 2023

Reporting Summary

Nature Portfolio wishes to improve the reproducibility of the work that we publish. This form provides structure for consistency and transparency in reporting. For further information on Nature Portfolio policies, see our Editorial Policies and the Editorial Policy Checklist.

Statistics

For all statistical analyses, confirm that the following items are present in the figure legend, table legend, main text, or Methods section.

n/a	Confirmed	
☐	☒	The exact sample size (*n*) for each experimental group/condition, given as a discrete number and unit of measurement
☐	☒	A statement on whether measurements were taken from distinct samples or whether the same sample was measured repeatedly
☐	☒	The statistical test(s) used AND whether they are one- or two-sided<br>*Only common tests should be described solely by name; describe more complex techniques in the Methods section.*
☒	☐	A description of all covariates tested
☒	☐	A description of any assumptions or corrections, such as tests of normality and adjustment for multiple comparisons
☐	☒	A full description of the statistical parameters including central tendency (e.g. means) or other basic estimates (e.g. regression coefficient) AND variation (e.g. standard deviation) or associated estimates of uncertainty (e.g. confidence intervals)
☒	☐	For null hypothesis testing, the test statistic (e.g. *F*, *t*, *r*) with confidence intervals, effect sizes, degrees of freedom and *P* value noted<br>*Give P values as exact values whenever suitable.*
☒	☐	For Bayesian analysis, information on the choice of priors and Markov chain Monte Carlo settings
☒	☐	For hierarchical and complex designs, identification of the appropriate level for tests and full reporting of outcomes
☒	☐	Estimates of effect sizes (e.g. Cohen's *d*, Pearson's *r*), indicating how they were calculated

Our web collection on statistics for biologists contains articles on many of the points above.

Software and code

Policy information about availability of computer code

Data collection	Matlab R2020b software and Micro-Manager 2.0beta were used to control microscopes for image acquisition.
Data analysis	BiofilmQ v0.2.2 was used to analyse biofilm images: https://drescherlab.org/data/biofilmQ/ .

For manuscripts utilizing custom algorithms or software that are central to the research but not yet described in published literature, software must be made available to editors and reviewers. We strongly encourage code deposition in a community repository (e.g. GitHub). See the Nature Portfolio guidelines for submitting code & software for further information.

Data

Policy information about availability of data

All manuscripts must include a data availability statement. This statement should provide the following information, where applicable:

- Accession codes, unique identifiers, or web links for publicly available datasets
- A description of any restrictions on data availability
- For clinical datasets or third party data, please ensure that the statement adheres to our policy

Transcriptome spatiotemporal heatmaps and heatmaps of microscopy-based measurements are available in a curated dataset explorer at https://drescherlab.org/data/swarm-transcriptome/. Transcriptome data that were used to generate the heatmaps are also available the National Center for Biotechnology Information Gene Expression Omnibus under the accession number GSE224332 (reviewer access code: ghadsicwzlalhwl, https://www.ncbi.nlm.nih.gov/geo/query/acc.cgi?acc=GSE224332). Files for viewing a 3D CAD model of the robotic sampling system are available on Zenodo (DOI: 10.5281/zenodo.8229225), and can be viewed with

the AutoDesk Inventor software. Coefficients of the spectral representation of each gene and physical property as well as pattern identification and multidimensional scaling coordinates used in Fig. 2 are available on Zenodo (DOI: 10.5281/zenodo.8355669). Metabolomics data are available on Zenodo (DOI: 10.5281/zenodo.8348257). Raw imaging data (1.86 terabyte in size) are available from the corresponding authors upon reasonable request, if infrastructure for transferring and storing these data are available.

Research involving human participants, their data, or biological material

Policy information about studies with human participants or human data. See also policy information about sex, gender (identity/presentation), and sexual orientation and race, ethnicity and racism.

Reporting on sex and gender	not applicable
Reporting on race, ethnicity, or other socially relevant groupings	not applicable
Population characteristics	not applicable
Recruitment	not applicable
Ethics oversight	not applicable

Note that full information on the approval of the study protocol must also be provided in the manuscript.

Field-specific reporting

Please select the one below that is the best fit for your research. If you are not sure, read the appropriate sections before making your selection.

☒ Life sciences ☐ Behavioural & social sciences ☐ Ecological, evolutionary & environmental sciences

For a reference copy of the document with all sections, see nature.com/documents/nr-reporting-summary-flat.pdf

Life sciences study design

All studies must disclose on these points even when the disclosure is negative.

Sample size	The sample size (n, corresponding to the number of independently performed replicas) for each experiment is indicated in the caption.
Data exclusions	No data were excluded.
Replication	Each experiment was replicated n times (and n is given in each figure for each experiment). Although the exact quantitative results differ between replicates, the qualitative results were the same, so that it is reasonable to state that the "replication was successful".
Randomization	There were many bacterial cells within each of the n replicates. Because of the large sample size for each replicate, a representative number of samples were collected for each replicate. There was no allocation of samples into experimental groups, beyond conducting independent biological replicates.
Blinding	Blinding of group allocation is irrelevant to our data analysis, because there was no allocation to experimental groups, beyond collecting n replicates, all of which were analyzed by software equally.

Reporting for specific materials, systems and methods

We require information from authors about some types of materials, experimental systems and methods used in many studies. Here, indicate whether each material, system or method listed is relevant to your study. If you are not sure if a list item applies to your research, read the appropriate section before selecting a response.

Materials & experimental systems

n/a	Involved in the study	
☒	☐	Antibodies
☒	☐	Eukaryotic cell lines
☒	☐	Palaeontology and archaeology
☒	☐	Animals and other organisms
☒	☐	Clinical data
☒	☐	Dual use research of concern
☒	☐	Plants

Methods

n/a	Involved in the study	
☒	☐	ChIP-seq
☒	☐	Flow cytometry
☒	☐	MRI-based neuroimaging
